# Cytoplasmic E2f4 forms organizing centres for initiation of centriole amplification during multiciliogenesis

**DOI:** 10.1038/ncomms15857

**Published:** 2017-07-04

**Authors:** Munemasa Mori, Renin Hazan, Paul S. Danielian, John E. Mahoney, Huijun Li, Jining Lu, Emily S. Miller, Xueliang Zhu, Jacqueline A. Lees, Wellington V. Cardoso

**Affiliations:** 1 Columbia Center for Human Development, Department of Medicine, Pulmonary Allergy Critical Care, Columbia University Medical Center, New York City, New York 10032, USA; 2 David H. Koch Institute for Integrative Cancer Research, MIT, Cambridge, Massachusetts 02139, USA; 3 State Key Laboratory of Cell Biology, Institute of Biochemistry and Cell Biology, Shanghai Institutes for Biological Sciences, Chinese Academy of Sciences, 320 Yueyang Road, Shanghai 200031, China

## Abstract

Abnormal development of multiciliated cells is a hallmark of a variety of human conditions associated with chronic airway diseases, hydrocephalus and infertility. Multiciliogenesis requires both activation of a specialized transcriptional program and assembly of cytoplasmic structures for large-scale centriole amplification that generates basal bodies. It remains unclear, however, what mechanism initiates formation of these multiprotein complexes in epithelial progenitors. Here we show that this is triggered by nucleocytoplasmic translocation of the transcription factor E2f4. After inducing a transcriptional program of centriole biogenesis, E2f4 forms apical cytoplasmic organizing centres for assembly and nucleation of deuterosomes. Using genetically altered mice and E2F4 mutant proteins we demonstrate that centriole amplification is crucially dependent on these organizing centres and that, without cytoplasmic E2f4, deuterosomes are not assembled, halting multiciliogenesis. Thus, E2f4 integrates nuclear and previously unsuspected cytoplasmic events of centriole amplification, providing new perspectives for the understanding of normal ciliogenesis, ciliopathies and cancer.

## Abstract (web summary)

Multiciliogenesis requires activation of transcriptional and protein assembly programs; however, the mechanisms that initiate the formation of these multiprotein complexes are unclear. Here the authors show that after inducing centriole biogenesis genes, the transcription factor E2f4 is required in the cytoplasm for assembly and nucleation of deuterosomes.

Cilia are microtubule-enriched structures that protrude from the surface of eukaryotic cells, appearing in different forms and with distinct biological roles^1^,^2^,^3^. Primary cilia act as sensory antenna and signalling hubs that transmit signals into the cell. In contrast, multicilia are motile structures that decorate the apical surface of epithelial cells in the respiratory and the reproductive tracts, ependyma and choroid plexus. Multiciliated cells play key roles in fluid movement and absorption^4^, as well as in organ defence by acting on mucociliary clearance^5^. Dysfunction of primary cilia or multicilia results in human ciliopathies, conditions associated with high morbidity and mortality^6^. Primary cilium appears in G0/G1 phase cells when the centrosome migrates to the cell surface, whereupon the mother centriole forms a basal body that gives rise to the cilium^7^,^8^. The process begins with formation of a complex between the centrosomal proteins Cep152 and Cep63 on the proximal side of the mother centriole followed by recruitment of Plk4 (polo-kinase 4), Sas6 (centriolar assembly protein), Centrin and other centriolar proteins, which create a cartwheel-like structure that nucleates the microtubule protuberances to form the cilium^9^,^10^,^11^,^12^,^13^. Primary cilia formation is entirely mother centriole-dependent, which replicates only a single daughter centriole during cell cycle. While the mother centriole pathway also contributes to multiciliogenesis, it is wholly insufficient for this role due to its inability to generate the large number of centrioles required for multicilia formation^6^,^14^. Classical electron microscopy studies of multiciliogenesis identified 40–80 micron electron–dense granular organelles, called fibrous granules (FGs), and numerous ring-like structures, the deuterosomes, present within and adjacent to FGs^15^,^16^,^17^,^18^. Subsequent studies showed that FG are enriched for Pcm1 (ref. 19), which was originally thought to be dispensable for multiciliogenesis based on RNAi knockdown^20^. Deuterosomes share many molecular similarities, but also key differences, with the mother centriole^2^. Many constituent proteins are conserved between the two structures, including Plk4, Cep152, Sas6 and Centrin^10^,^12^,^21^,^22^. In contrast, Cep63—of the mother centriole complex—is replaced by a related protein Deup1 (or Ccdc67) for deuterosome formation^21^. Deuterosome is crucial for procentriole multiplication. A single deuterosome can hold multiple procentrioles, and efficiently produce the few hundreds centrioles required for multiciliogenesis. The existence of this deuterosome-dependent (DD) process allows centriole amplification and the development of multiciliated cell uncoupled from cell cycle control.

There is evidence that multiciliogenesis is triggered by induction of an E2f4-dependent transcriptional program of centriole biogenesis in multiciliated cell precursors^23^,^24^. E2f4 along with other E2f family members are transcription factors best known for regulating cell proliferation^25^,^26^,^27^,^28^. Notably, E2f4 has been recently shown to mediate the transcriptional responses of Multicilin (*Mcidas*), a human ciliopathy-associated gene encoding a well-conserved regulator of multiciliogenesis in *Xenopus*^24^ and mice^23^,^29^. Although these studies show activation of a panel of multiciliogenesis genes, including Deup1, it is still unknown what mechanism leads to initiation of deuterosome assembly in multiciliated precursor cells following the activation of the centriole biogenesis transcriptional program. Some studies propose that FGs seed this process^15^,^16^,^18^, but others argue that deuterosomes assembly is spontaneous^2^,^21^ or caused by other, yet to be determined, proteins^22^.

Here, we show that this mechanism is crucially dependent on the nucleocytoplasmic translocation of E2f4. We provide evidence that cytoplasmic E2f4 forms organizing centres for assembly and nucleation of deuterosomes to initiate large-scale centriole amplification in epithelial progenitors.

## Results

### E2f4 undergoes nucleocytoplasmic shift during multiciliogenesis

**Figure 1:**
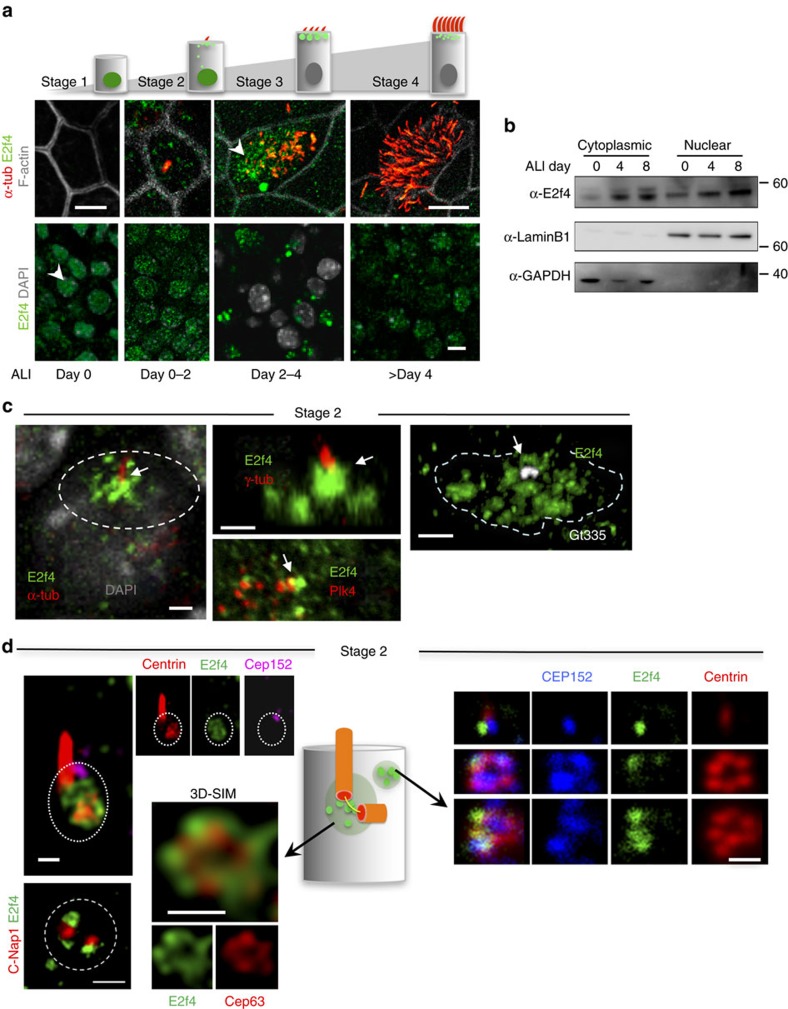
E2f4 undergoes nucleocytoplasmic translocation to form apical aggregates with centriole biogenesis components during multiciliogenesis. (**a**) Immunofluorescence (IF)-confocal imaging depicting apical and basal aspects of adult airway progenitors at distinct stages of multiciliogenesis in air-liquid interface (ALI) cultures (acetylated α-tub, E2f4; top panels, cells outlined by F-actin; bottom panels: DAPI: nuclear staining). Arrowheads depict E2f4 subcellular localization along with the markers indicated. (**b**) Western blotting of E2f4 nuclear and cytoplasmic fractions of ALI culture extracts at days 0, 4, 8: E2f4 accumulation in cytoplasm during differentiation (controls for cellular fractionation: nuclear LaminB1, cytoplasmic GAPDH). (**c**) Confocal analyses, maximum projection view, stage 2 cells: E2f4 apical aggregates (circled areas, arrows) underneath primary cilium marked by acetylated α-tub, γ-tub, Gt335 or in adjacent regions; partial overlap of E2f4 with Plk4 (arrow). (**d**) IF imaging and diagram depicting cytoplasmic E2f4 at the centrosomal region (circled area in left panel, associated with c-Nap1, centrin, Cep152) forming ring-like structure with Cep63 (3D-SIM) and at non-centrosomal regions (right panel) forming similar structures with Cep152 and centrin in stage 2 cells. Bars: **a**,**c**,**d**=10, 2.5, 0.5 μm, respectively.

To gain initial insights into the ontogeny of E2f4 in differentiating epithelial cells, we mapped its expression pattern during step-wise initiation of multiciliogenesis in airway epithelial progenitors using a well-established model of air–liquid interface (ALI) culture^30^,^31^,^32^,^33^. In this assay, adult airway epithelial progenitors isolated from adult trachea are expanded to confluence and exposed to an ALI, which triggers differentiation, identified through a series of well-defined stages. Indirect immunofluorescence (IF) for E2f4 and confocal imaging of confluent multipotent progenitors prior to ALI induction (Stage 1, ALI day 0) showed nuclear-specific localization of E2f4 (Fig. 1a, Supplementary Fig. 1a). However, once the cells were exposed to an ALI, E2f4 underwent a striking nuclear-to-cytoplasmic translocation to later become essentially cytoplasmic in multiciliated cells (Fig. 1a). Small apical cytoplasmic E2f4 granules (<1 μm) were initially seen in monociliated cells (Stage 2; ALI day 0–2). Larger apical E2f4 aggregates (>1 μm diameter) then became evident in ∼80% of the cells as multicilia started to form (Stage 3; ALI day 2–4) (Fig. 1a, Supplementary Fig. 1a,b). At a later stage, smaller E2f4 granules (<1 μm) were again observed in mature multiciliated cells bearing longer cilia (Stage 4; after ALI day 4). Importantly, we found that E2f4 cytoplasmic localization occurred essentially in cells undergoing multiciliogenesis, but not in secretory progenitors, which predominantly retained nuclear E2f4 (Supplementary Fig. 1c). The subcellular localization of E2f4 was further demonstrated by cell fractionation and western blotting (Fig. 1b, Supplementary Fig. 10). We extended our analyses to examine the E2f4 subcellular localization during airway differentiation *in vivo*. Immunostaining of developing (E12.5–E18.5) and adult murine lungs showed apical accumulation of E2f4 clearly apparent at E16.5, coincident with the emergence of multicilia (Supplementary Fig. 1d). Furthermore, IF analysis of adult human lung sections detected strong cytoplasmic E2F4 in multiciliated cells marked by acetylated α-tubulin in stark comparison to the lower level nuclear E2F4 staining in the airway progenitor basal cells (Supplementary Fig. 1e). Thus, our data suggest that the E2f4 nucleocytoplasmic shift during multiciliogenesis is evolutionary conserved between mouse and human airways.

### E2f4 localizes to areas of procentrioles initiation

To gain insights into the cellular events mediated by cytoplasmic E2f4, we coupled IF with confocal and super-resolution three-dimensional-structured illumination microscopy (3D-SIM) and examined how the distribution of E2f4 protein correlated with the appearance of early markers of ciliogenesis. Already early in stage 2 cells, E2f4 cytoplasmic signals were clearly detected at two distinct apical sites: beneath primary cilia, partially overlapping with the ciliary markers acetylated α-tubulin, γ-tubulin and glutamylated tubulin (Gt335), and in its immediate neighbourhood as multiple small foci or granules (Fig. 1c,d). At the primary cilium 3D-SIM revealed E2f4 forming ring-like structures with Cep63 in a region enriched in Cep152, Plk4 and C-Nap1 (centrosomal protein Cep250), which marks sites of procentriole nucleation in parental centrioles^34^. The multiple other apical E2f4 foci outside the centrosomal region, also showed ring-like structures enriched in Cep152, centrin and the deuterosome protein Deup1, but not Cep63 (Fig. 1d, Supplementary Fig. 2a,b). Thus, at the onset of multiciliogenesis both sites of E2f4 expression correlated with markers of procentrioles initiation. We reasoned that cytoplasmic E2f4 foci could be active sites of centriole biogenesis in these epithelial progenitors. This was reinforced by our analysis of stage 2–3 cells showing that cells expressing Plk4, a crucial kinase recruited by Cep152 to initiate centriogenesis^12^,^21^, were mostly double-labelled with E2f4 (80%) or with those cells expressing both E2f4 and Cep152 (∼65–70%) (Supplementary Fig. 2a).

### E2f4-Pcm1 form organizing centres for centriole biogenesis

**Figure 2:**
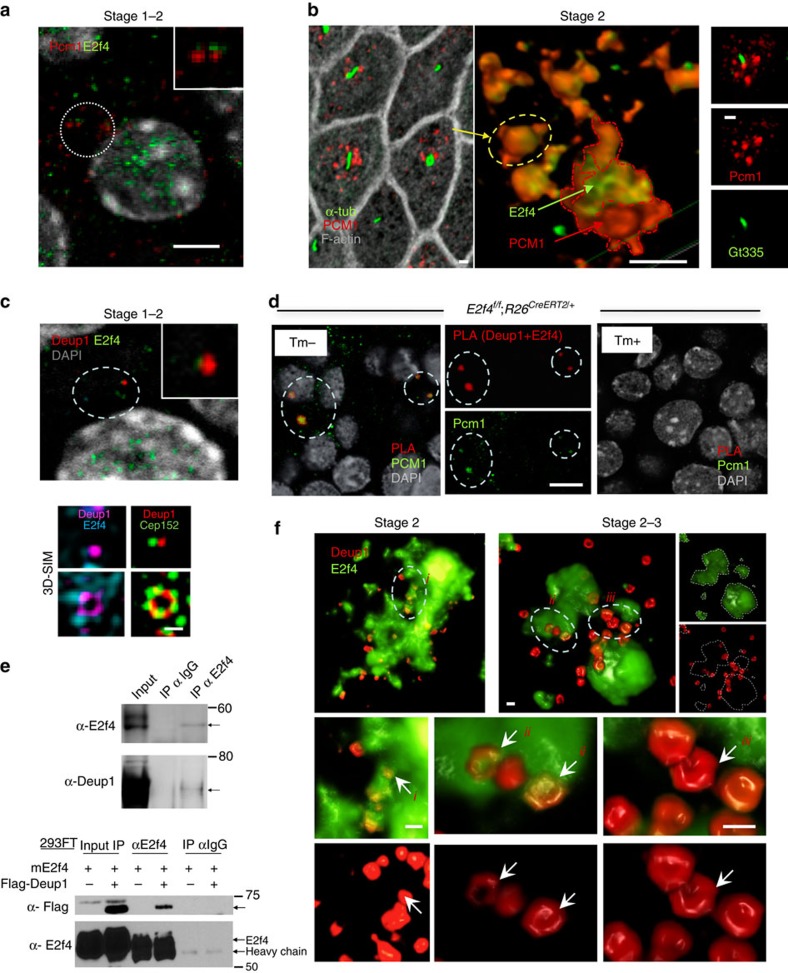
Deuterosomes assemble in cytoplasmic E2f4-Pcm1 granules. (**a**) E2f4-Pcm1 IF confocal image, stage1-2 transition during E2f4 nucleocytoplasmic shift: Pcm1-E2f4 signals adjacent but still not overlapping (inset, enlarged from circled area). (**b**) Left panel: confocal image stage 2 cells showing apical Pcm1-labelled granules nearby primary cilia (acetylated α-tub); middle panel: 3D-SIM: Pcm1-E2f4 colocalization (circled area representative of apical granule on left): E2f4 at the core of Pcm1 granules); right panel: confocal image depicts Pcm1 underneath and nearby Gt335-labelled primary cilium. See also Supplementary Movie 1. (**c**) IF confocal (upper panel) and 3D-SIM (lower panel) of stage 1–2 cells: E2f4 nucleocytoplasmic translocation and initial association with Deup1 (inset, enlarged from circled area). Assembly of Deup1, Cep152 and cytoplasmic E2f4 into deuterosomes. (**d**) Proximity ligation assay (PLA): Deup1+E2f4 proximity signal overlapping with Pcm1 (left and split panels: circled areas in ALI day3 from *E2f4*^*f/f*^; *R26*^*CreERT2/+*^ no Tm administration); specificity of signals confirmed in E2f4-deficient cells (right panel; Tm-treated *E2f4*^*f/f*^; *R26*^*CreERT2/+*^ cultures; see also Supplementary Fig. 4a). (**e**) Co-immunoprecipitation (coIP) assays: binding of endogenous (top) or exogenous (bottom) E2f4 to Deup1 in ALI day 3 (stage 2) or Deup1-Flag in 293FT homogenates, respectively (see methods). (**f**) Deup1-E2f4 IF, 3D-SIM reconstruction of stage 2 cells (top left) and cells transitioning to stage 3 (top right). Small Deup1 dots assemble into rings inside E2f4 granules but, later, more mature deuterosomes with larger rings locate at the periphery or outside the E2f4 granules. Arrows *i,ii,iii*: representative Deup1 rings from circled areas enlarged in lower panels shown by split channel (E2f4) or double Deup1-E2f4 labelling (less to more mature deuterosomes from left to right). Bars: **a**–**d**,**f**=5, 1, 0.5, 5, 0.5 μm, respectively.

Interestingly, the timing of appearance and spatial distribution of cytoplasmic E2f4 was reminiscent of that described for FG that form during initiation of large-scale centriole amplification^15^,^19^,^35^. Indeed, as multipotent progenitors transitioned to stage 2 and E2f4 protein started undergoing nucleocytoplasmic shift, Pcm1 could be detected as discrete punctate cytoplasmic signals closely associated with E2f4. Both signals subsequently overlapped to form small apical aggregates around and underneath monocilia (marked by acetylated α–tubulin and Gt335; Fig. 2a,b). By stage 2–3 confocal analysis demonstrated E2f4-Pcm1 overlap in aggregates of variable sizes. Colocalization was demonstrated by quantification of signal intensities in double-labelled sections, which showed the highest E2f4 signal between Pcm1 peaks (Supplementary Fig. 2c). 3D-SIM further confirmed that E2f4 signals are surrounded by Pcm1, clearly establishing the E2f4’s location at the core of Pcm1-containing FG (Fig. 2b; Supplementary Fig. 2c; Supplementary Movie 1). The enrichment in centriole biogenesis components in E2f4-Pcm1 granules suggested a role as organizing centres for nucleation of centrioles.

### Deuterosomes assemble in cytoplasmic E2f4-Pcm1 granules

To further understand the involvement of cytoplasmic E2f4 in centriole amplification, we examined assembly of deuterosomes in our system. Deup1 specifically label deuterosomes, the structures that support large-scale (*de novo*) centriole amplification for multiciliogenesis^2^,^21^. Deup1 is a crucial regulator of deuterosome assembly and known to interact with Cep152 and recruit Plk4 to activate centriole biogenesis^21^,^22^. Confocal and 3D-SIM imaging of epithelial progenitors transitioning to Stage 2 cells showed Deup1 signals associated with emerging cytoplasmic E2f4 and assembling into characteristic ring-like structures with Cep152. Notably, small cytoplasmic E2f4 granules (<1 μm) were clearly localized around the Deup1 rings (Fig. 2c). Deup1-E2f4-Cep152 co-immunostaining and profile analysis of confocal sections demonstrated extensive signal overlap as more apical granules accumulated later in Stage 2 cells. This was prominent in the larger apical granules of stage 2–3 cells, suggesting these to be sites of active deuterosome assembly (Supplementary Fig. 2d). We then further examined the association between E2f4 and Deup1 in our cells by performing immunoprecipitation and western blot assays in extracts from adult ALI airway epithelial cultures, and in 293FT cell lines transfected with E2f4 and Deup1. These analyses revealed a clear interaction between E2f4 and Deup1 for both the endogenous and ectopically expressed proteins (Fig. 2e, Supplementary Fig. 10). We also gathered biochemical evidence of E2f4 interaction with Sas6, another centriole biogenesis core component expressed in these cells (Supplementary Figs 2e,f and 10). Next, we probed the intimate association of E2f4 and Deup1 during multiciliogenesis by performing Proximity Ligation Assay (PLA) in ALI cultures from *E2f4*^*f/f*^; *R26*^*CreERT2/+*^ adult airway progenitors (Tm-treated cultures as negative controls). This revealed strong E2f4-Deup1 PLA signals specifically in E2f4-sufficient, but not E2f4-deficient cells (Fig. 2d, Supplementary Fig. 4a). Importantly, we found that E2f4-Deup1 PLA signals overlapped with a subset of Pcm1 foci demonstrating that all three proteins co-exist in close proximity. Collectively, our data show that cytoplasmic E2f4 is present in complexes with two known deuterosome components, Deup1 and Sas6. These complexes appear to occur in the same location as the Pcm1-containing FG.

To more precisely define the spatial–temporal relationship between FGs and deuterosomes during centriole amplification, we performed triple IF staining for Pcm1, Deup1 and the centriolar marker Centrin. Quantitative analysis showed an increasing number of deuterosomes per cell and increasing Centrin-decorated deuterosomes as cells differentiate during stages 1–3 (Supplementary Fig. 3). Interestingly, while in immature cells at stage 2 the vast majority of Deup1 signals were found in Pcm1-enriched areas, the Deup1-Pcm1 co-localization decreased later as cells matured and underwent centriole amplification (Supplementary Fig. 3, Supplementary Movie 3–5). Indeed, at later stages centrin-decorated deuterosomes were increasingly more frequent outside Pcm1 areas (Supplementary Fig. 3a,b,g). Moreover, 3D-SIM imaging of Deup1-E2f4 double-labelled cells and assessment of deuterosome size showed that mature deuterosomes, harbouring compact rings with larger diameters^21^, were most frequently found at the periphery or outside the E2f4-rich regions (Fig. 2f, *i*,*ii,iii* in right panel and in Supplementary Fig. 4b,c; Supplementary Movie 2). The abundant centrin labelling outside the E2f4-Pcm1 regions and dissociated from deuterosomes in stage 4 cells suggested a lesser role for E2f4 after centriole amplification. Our observations are consistent with the idea that deuterosomes arise and start nucleating in Pcm1-E2f4 enriched areas, as formerly proposed for FG^16^,^19^,^35^.

### Conserved E2f4’s role in centriole biogenesis gene transcription

We examined whether key Multicilin-E2f4 targets identified during *Xenopus* multiciliogenesis^24^ were similarly regulated by endogenous E2f4 in our system. Mice with systemic E2f4 deficiency were previously shown to be unable to form multicilia in the respiratory epithelium^23^. However further insights into the cellular events and targets regulated by E2f4 were limited by neonatal lethality due to multiple defects^23^. To overcome this limitation, we generated *E2f4*^*f/f*^; *R26*^*CreERT2/+*^ mice to delete *E2f4* selectively in epithelial progenitors either *in vivo* using a *Shh*^*Cre*/+^ line that induces recombination early in the lung epithelium *(E2f4*^*f/f*^*;Shh*^*Cre/+*^, herein *E2f4*^*cnull*^) or in vitro in adult ALI cultures upon 4-hydroxytamoxifen treatment (Tm)^36^. Results from both approaches showed that E2f4 deletion does not prevent primary cilia formation but abolishes multiciliated cells (Supplementary Fig. 5a and 6a–d). We then performed whole-genome expression profiling of adult control and Tm-treated *E2f4*^*f/f*^; *R26*^*CreERT2/+*^ airway epithelial progenitors prior to and during initiation of multiciliogenesis (ALI days 0, 2 and 4). Remarkably, this confirmed the enrichment in centriole biogenesis genes and extensive overlap with the E2f4 targets formerly reported in *Xenopus*^24^. Clustering analysis and qPCR analyses revealed *Deup1*, *Ccno*, *Myb* and others in this group (*Deup1* cluster) significantly reduced at ALI day 0, while *Plk4*, *Sas6*, *Stil* mRNAs (*Plk4* cluster) decreased at ALI day2 (Supplementary Fig. 5). A comprehensive characterization of the E2f4 transcriptional targets from these studies will be reported elsewhere. Analysis of E18.5 lungs and adult ALI cultures demonstrated the loss of Foxj1, β-tubulin expression and disruption of multiciliogenesis in E2f4-deficient epithelium. Importantly, IF showed none of the large apical aggregates of Deup1, Cep152, Pcm1 present in E2f4-sufficient cells (Supplementary Fig. 6).

### Multiciliogenesis depends on E2f4 nucleocytoplasmic localization

**Figure 3:**
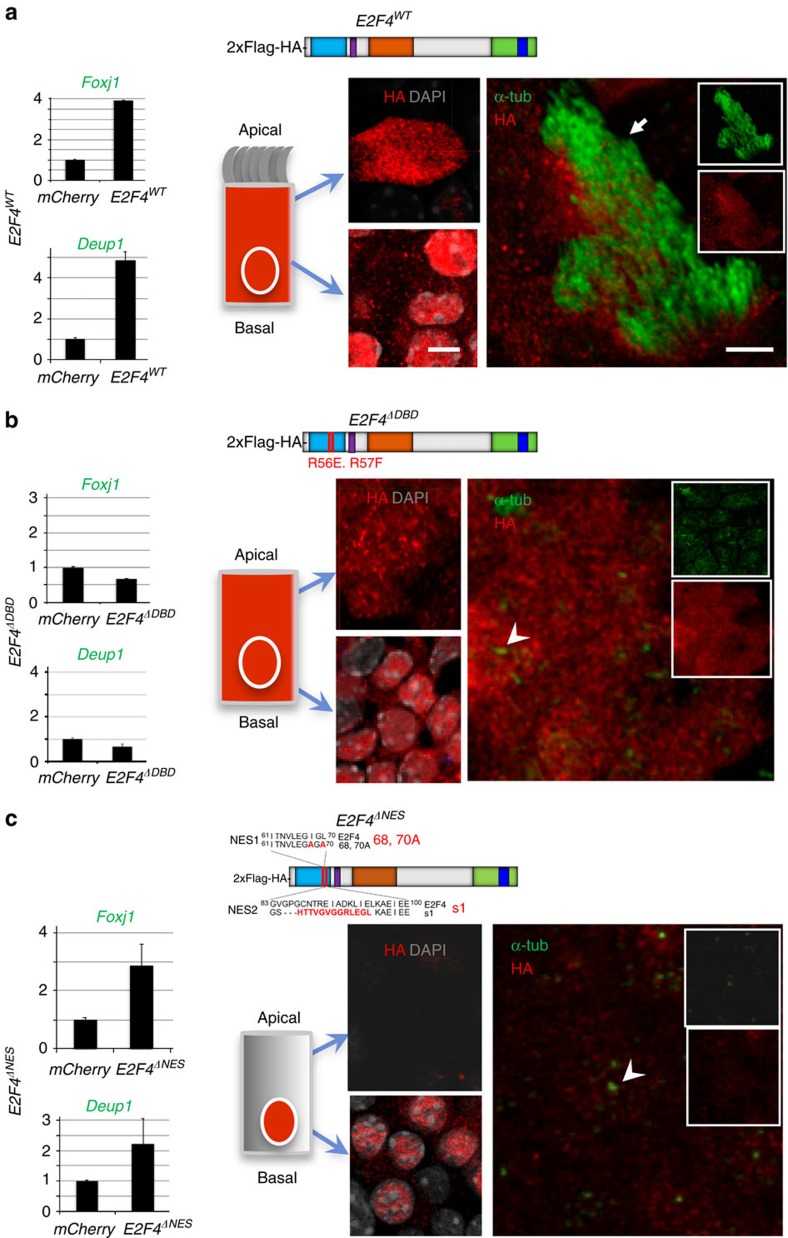
E2f4 nucleocytoplasmic shift is crucial for multicilia formation. Lentiviral-mediated transduction of HA-tagged E2F4 (diagrams: *E2F4*^*WT*^*, E2F4*^*ΔDBD*^*, E2F4*^*ΔNES*^) or mCherry control constructs in *E2f4*^*f/f*^;*R26*^*CreERT2/+*^ airway progenitors treated with Tm (Tamoxifen). Left panels: qPCR of ALI day6: increased *Foxj1* and *Deup1* mRNAs by *E2F4*^*WT*^ or *E2F4*^*ΔNES*^ or but not *E2F4*^*ΔDBD*^, compared to *mCherry.* Bars are mean (+s.e.m.). (**a**–**c**) Right panels: Immunofluorescence/confocal imaging: HA, acetylated α-tubulin, DAPI (diagram, apical and basal aspects). HA signals in both nucleus and cytoplasm in *E2F4*^*WT*^ and *E2F4*^*ΔDBD*^ but only in nuclei of *E2F4*^*ΔNES*^. Rescue of multiciliogenesis (arrow) in E2f4-deficient cells by transduction of *E2F4*^*WT*^ but not *E2F4*^*ΔDBD*^ or *E2F4*^*ΔNES*^ (arrowheads: monocilia). Bar in **a**=5 μm.

**Figure 4:**
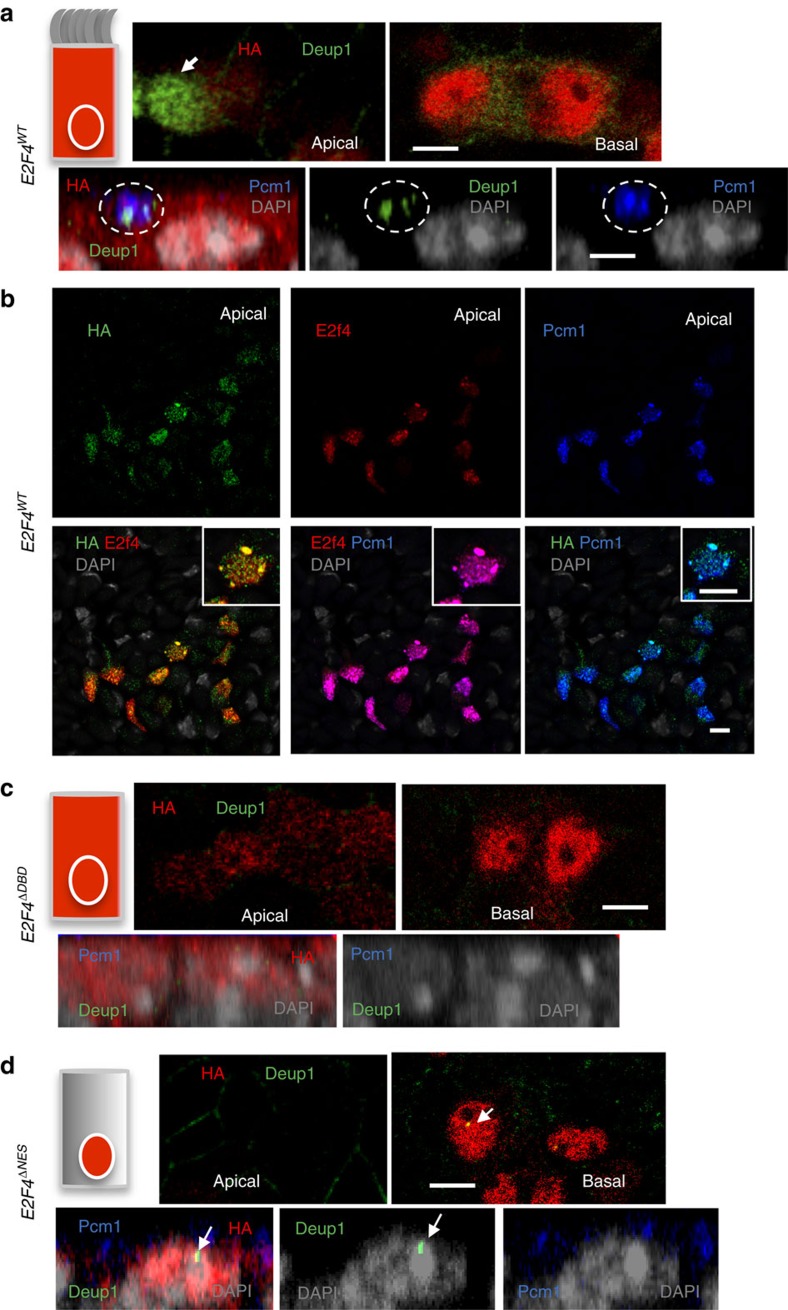
Cytoplasmic E2f4 is required for apical accumulation of Deup1 and Pcm1. (**a**) Strong apical cytoplasmic induction of Deup1 in Tm-treated *E2f4*^*f/f*^;*R26*^*CreERT2/+*^ transduced with *E2F4*^*WT*^ (arrow in top panel: ALI day6 cells); HA-Deup1-Pcm1 aggregates confirmed in side projection views at ALI day2 (circled area in bottom panel). (**b**) *E2F4*^*WT*^-transduced cells express HA, E2f4, and Pcm1 in overlapping patterns in large and smaller cytoplasmic granules (single channels and double-labelled low magnification panels; inset: representative double-labelled cell at high magnification; DAPI). (**c**) *E2F4*^*ΔDBD*^-transduced cells: HA expression in nucleus and cytoplasm but no Deup1 or Pcm1 signals. (**d**) *E2F4*^*ΔNES*^-transduced cells: HA expression selectively nuclear; Deup1 mislocalized in nuclei (arrow) and unable to form apical aggregates with Pcm1. All bars=5 μm.

The observations above supported a broader role for E2f4 in multiciliogenesis, beyond that originally associated with its transcriptional activity^24^ and not yet tested in functional studies. These results, however, could not provide us with direct insights into a role for cytoplasmic E2f4 as they could be ascribed to disruption of the transcriptional program of centriole biogenesis. To resolve this issue, we generated E2F4 mutants that discriminate nuclear from cytoplasmic functions, and compared their ability to rescue multiciliogenesis in E2f4-deficient airway progenitors. Lentivirus constructs were generated carrying point mutations that specifically disrupt the E2f4’s DNA binding domain resulting in a transcriptionally inactive form (*E2F4*^*ΔDBD*^), or that disrupt its nuclear export signal, preventing cytoplasmic localization (*E2F4*^*ΔNES*^)^37^. Flag-HA-tagged versions of *E2F4*^*ΔDBD*^, *E2F4*^*ΔNES*^ or controls mCherry and wild type (*E2F4*^*WT*^) were transduced in Tm-treated *E2f4*^*f/f*^; *R26*^*CreERT2/+*^ adult airway progenitors and multiciliogenesis was assessed in ALI cultures. qPCR analyses confirmed that both *E2F4*^*ΔNES*^ and *E2F4*^*WT*^ were indeed transcriptionally active, unlike *mCherry* and *E2F4*^*ΔDBD*^ mutants, which were unable to induce expression of the E2f4 target genes *Deup1* and *Foxj1* (Fig. 3, left panels). HA immunofluorescence analyses confirmed the nucleocytoplasmic localization of all mutants (Fig. 3). Expected cytoplasmic HA signal was particularly evident in the cells transduced with the *E2F4*^*WT*^construct, which showed large and small apical granules double-labelled with HA and E2f4 antibodies (Fig. 4b). Importantly, *E2F4*^*ΔNES*^-transduced cells displayed HA signals highly restricted to the nucleus, supporting reliability of our targeting approach (Figs 3c and 4d).

Analysis of *E2F4*^*WT*^ ALI day 6 cultures showed robust rescue of multiciliogenesis with HA-labelled cells expressing Foxj1, centrin and acetylated α–tubulin-labelled structures compatible with multicilia (Fig. 3a; Supplementary Fig. 7). This contrasted with the inability to form multicilia in the cultures transduced with mCherry (control) and the transcriptionally inactive *E2F4*^*ΔDBD*^. Notably, although *E2F4*^*ΔNES*^ was present in the nucleus and able to induce *Foxj1 mRNA*, no Foxj1 immunostaining was detected by day 6 in cultures, in agreement with the *E2F4*^*ΔNES*^ failure to initiate multiciliogenesis in E2f4-deficient cells (Fig. 3b,c, Supplementary Fig. 7).

### Cytoplasmic E2f4 is required for deuterosome assembly

Consistent with the presence of cytoplasmic HA-E2F4 and the rescue of multiciliogenesis, we found accumulation of apical Deup1 granules in *E2F4*^*WT*^–transduced cells (Fig. 4a). Thus, *E2F4*^*WT*^ expression in the nucleus induced *Deup1* and its ability to localize to the cytoplasm allowed assembly of deuterosomes. In addition, *E2F4*^*WT*^ supported induction and accumulation of Pcm1 in the cytoplasm of these cells. The HA-regions were often associated with Pcm1 staining in a pattern reminiscent of the FG that we identified *in vivo* (Fig. 4a,b). This pattern, however, was not always clear due to the high expression levels of the HA-tagged constructs, making HA to often appear more evenly distributed in the cytoplasm. As expected, no Deup1 or Pcm1 signals were detected in *E2F4*^*ΔDBD*^ cells (Fig. 4c). Remarkably, although *E2F4*^*ΔNES*^ was competent to induce *Deup1*, its inability to undergo nucleocytoplasmic shift resulted in inappropriate localization of Deup1 protein. In the absence of cytoplasmic E2F4, Deup1 was unable to form the characteristic apical cytoplasmic aggregates. Instead, Deup1 accumulated in the nucleus as early as ALI day 2 or could be found less frequently non-aggregated in the cytoplasm (Fig. 4d; Supplementary Fig. 8). Morphometric assessment of the subcellular distribution of Deup1 revealed nuclear signals in nearly 50% of ALI day6 *E2F4*^*ΔNES*^–transduced cells. This was in sharp contrast with *E2F4*^*WT*^, in which nuclear Deup1 was found in∼5% of the population (Supplementary Fig. 8c). Moreover, in the absence of cytoplasmic E2F4 there was no evidence of the Pcm1-containing apical granules or nucleating centrioles in the *E2F4*^*ΔNES*^ cells (Fig. 4d; Supplementary Fig. 7c).

**Figure 5:**
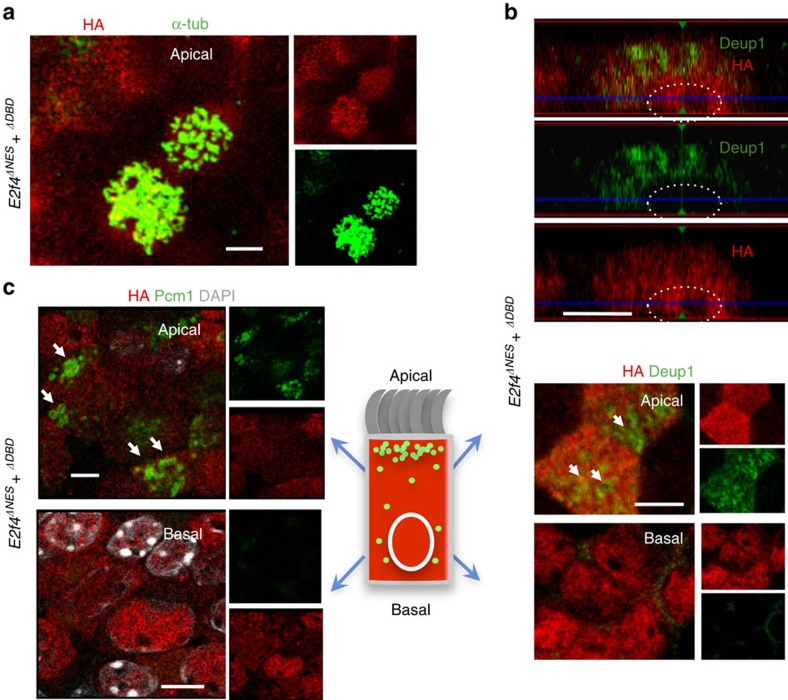
Restoration of nuclear and cytoplasmic E2f4 function rescues deuterosome formation and multiciliogenesis in E2f4-deficient cells. Co-transduction of *E2F4*^*ΔDBD*^ and *E2F4*^*ΔNES*^ in E2f-deficient cells; IF/confocal imaging. (**a**) Multicilia formation (acetylated α-tubulin) in HA-labelled cells. (**b**) Induction of multiple Deup1 apical aggregates (top: side projection view; bottom: apical and basal view, arrows). (**c**) Formation of apical HA-Pcm1 granules (arrows). All bars=5 μm.

At last, we investigated the co-requirement of nuclear and cytoplasmic E2F4 to assemble deuterosomes and initiate multiciliogenesis. Thus, we transduced simultaneously the two mutant constructs that failed individually to rescue the E2f4-deficient phenotype. Lentiviral-mediated cotransduction of *E2F4*^*ΔNES*^ and *E2F4*^*ΔDBD*^ in *E2f4* null cells resulted in transcription and apical targeting of Deup1, formation of Pcm1-containing granules and multicilia formation (Fig. 5). Taken together, we conclude that the multiciliogenesis program is crucially dependent on both nuclear and cytoplasmic functions of E2f4.

## Discussion

Here we provide evidence of an unsuspected role for cytoplasmic E2f4 as a core component of apical multiprotein complexes crucial for multiciliogenesis. The close association of E2f4 with Pcm1, as well as the timing and spatial distribution in airway progenitors strongly suggest that cytoplasmic E2f4-Pcm1 aggregates represent the FG classically described during multiciliogenesis. E2f4 is then likely the FG’s most relevant component, since others, such as Pcm1 and Bbs4 (Bardet–Biedl syndrome 4) have been shown not to be essential for multicilia formation^20^.

Our data support a model in which cytoplasmic E2f4 acts as the organizing centre for the recruitment and accumulation of early regulators of centriole biogenesis, to enable assembly and nucleation of deuterosomes (Supplementary Fig. 9). Thus, E2f4 acts in a sequential manner to link two distinct but interrelated processes in multiciliogenesis, first by inducing a transcriptional program of centriole biogenesis and then by promoting assembly of the resulting protein products in the cytoplasm to initiate centriole amplification.

These observations offer a new paradigm to understand the mechanisms that regulate multiciliogenesis in the lung and potentially other organs, such as the brain and reproductive tract, where multiciliated cells are abundant. They can also illuminate mechanisms of pathogenesis of ciliopathies and cancers in these organs.

## Methods

### Mouse strains and genotyping

The BAC clone 104J05 containing the *E2f4* genomic locus (129S6/SvEvTac genomic library; RPCI-22, BACPAC Resource Center, Children’s Hospital Oakland Research Institute) was used for generation of the targeting construct via recombineering using standard procedures (http://redrecombineering.ncifcrf.gov/)^38^. A DNA fragment harbouring a *FRT* flanked *PGKEM7neobpA* positive selection cassette containing one *loxP* sequence and 50 bp of homology to sequences in the first intron of *E2f4* at each end was generated by PCR (Expand High Fidelity PCR System, Roche) from PL451 and integrated into the *E2f4* genomic locus clone via recombineering (reagents provided by Drs Neal Copeland and Nancy Jenkins, NCI). Subsequently the plasmid pBR.DT-A (Addgene #35955) containing a diphtheria toxin A negative selection cassette was amplified using primers containing 50 bp of homology to sequences located 4.2 kb upstream and 5.8 kb downstream of the first exon of *E2f4* and the targeted locus was transferred into this vector via gap repair. The second *loxP* sequence was inserted into a *Kpn1* site located in intron four using standard cloning procedures. All modifications and the exons within the targeting vector were verified by DNA sequencing. Primer sequences are available on request. Following linearization the targeting vector was electroporated into v6.5 hybrid (C57BL/6 x 129S4/Jae) ES cells and DNA isolated from G418 resistant colonies was screened for homologous recombination by Southern blotting using probes 5′ and 3′ to the targeted locus and standard procedures. ES cells containing correctly targeted loci were additionally screened using a probe to the *neo* cassette to screen out clones carrying additional integrations of the targeting vector. Out of 223 clones picked, 8 were correctly targeted. C57BL/6 blastocysts were injected with correctly targeted ES cells and transplanted into pseudopregnant CD1 mice to generate chimeras. Chimeras were crossed with C57BL/6 females and following determination of germline transmission by Southern blotting. Mice heterozygous for the targeted allele were crossed with mice expressing FLPe recombinase from the *ROSA* locus (Jackson Laboratories, stock number 003946) to delete the *neo* cassette and generate a conditional floxed allele of *E2f4*, designated as *f.* In this allele Cre mediated recombination would delete exons 2 through 4, which is predicted to generate a null allele of *E2f4*. Genotyping was performed by PCR using the following primers for the *E2f4* conditional allele: F4cC, gccattaagcctcagctctgtctgg and F4cU, gtgcaccctgagatgtttagtctgg resulting in a 200 bp product for the wild-type allele and a 293 bp product for the conditional, floxed, allele. F4cC and a separate primer, ctggaacttgcaatgtagacaagg were used to detect the locus following recombination by Cre recombinase (244 bp product). The *ZsGreen1* Cre recombinase reporter allele^39^ and the *Shh-Cre* allele were purchased from Jackson Laboratories (stock numbers 007906 and 005622); the *Rosa26-CreERT2* allele^40^ was provided by Tyler Jacks' laboratory at MIT (NCI Mouse Repository stock number 01XAB). Mice were maintained on a mixed C57BL/6 × 129Sv background. All animal procedures and experiments were approved by the Institutional Animal Care and Use Committees at Columbia University and MIT.

### Air–liquid interface culture of airway epithelial progenitors

Airway epithelial progenitors were isolated from adult tracheas of wild type mice or E2f4 mouse mutants and cultured using well-established protocol (see Methods)^41^,^42^. For the functional analysis of E2f4 in ALI cultures we used adult *E2f4*^*+/+*^*, E2f4*^*+/f*^*; R26*^*CreERT2/LSLZsGreen1*^ *or E2f4*^*f/f*^*; R26*^*CreERT2/LSLZsGreen1*^ mice, as well as mice lacking the *R26LSLZsGreen1* allele. Briefly, after treatment with 0.5% pronase overnight, cells were cultured on collagen1-coated Transwell dishes (Corning) under submerged conditions in media that allowed expansion of airway progenitors^42^ until confluence (7 days). The ALI was established by removing media from the upper chamber of Transwell (Day 0 ALI) and culturing cells in differentiation media (mTEC/serum free, RA media)^42^ up to 8 days (Day 8 ALI). Treatment with 1 μM 4-hydroxytamoxifen (Tm) from day −5 to day 0 was used to induce Cre mediated recombination^36^. The efficiency of recombination was analysed by qPCR, IF for E2f4 and expression of ZsGreen1, where appropriate.

### E2F mutant constructs and lentiviral-mediated gene transduction

Lentiviral vectors were constructed to express 2 × Flag-HA-tagged wild-type E2F4, nuclear export signal mutant (ΔNES 68, 70A+E2F4 s1)^37^ or a DNA binding domain mutant (ΔDBD, encoding two amino acid replacements in the DNA recognition helix, R56E, R57F) for rescue experiments. Briefly, we amplified Not1-2 × Flag-HA-*E2F4* WT-BamH1 and Not1-2 × Flag-HA-*E2F4* NES (68, 70A+E2F4s1)-BamH1 PCR fragments by polymerase chain reaction (PCR) using pCMV-*E2F4* WT^43^ or pcDNA-*E2F4* NES (68, 70A,+E2F4s1) as templates and the primer sets of Not1-2 × Flag-HA-Forward primer: 5′gtcactGCGGCCGCACCGGTTAACccaccatgGACTACAAAGACCATGACGGTGATTATAAAGATCATGACATCGATTACagtTacccatacgacgtcccagactacgctATGGCGGAGGCCGGG-3′, and stop codon-BamH1-Reverse primer: 5′-tcactGGATCCACGCGTTTAACtcaGAGGTTGAGAACGGCACATC-3′. This PCR fragment was subcloned into the BamH1/Not1 sites of pHAGE-EF1a-Luciferase-W vector. Subsequently, we amplified Not1-2 × Flag-HA-E2F4 ΔDBD (R56E, R57F)-BamH1 PCR fragment by PCR-ligation mutagenesis^44^ with pHAGE-2 × Flag-HA-*E2F4* WT–w vector as the template and the primers which include R56E, R57F mutation: Forward 5′-CGCCAGAAGgagttcATTTACGACATTACCAATGTTTTGGAAGGT-3′, Reverse 5′-AGCTGTACGCCAGAAGgagttcATTTACGACA-3′. PCR products were generated using pHAGE-2 × Flag-HA-*E2F4* WT-w vector as the template and the following primer sets: Not1-2 × Flag-HA-Forward primer with R56E, R57F mutation-Reverse primer, and R56E, R57F mutation-Forward primer with stop codon-BamH1-Reverse primer. To obtain the Not1-2 × Flag-HA-*E2F4* ΔDBD (R56E, R57F)-BamH1 PCR fragment, we subsequently performed PCR with the mixture of these PCR products (1:1) as the template, and the primer sets of Not1-2 × Flag-HA-Forward primer and stop codon-BamH1-Reverse primer. The PCR fragment was subcloned into the pHAGE-EF1a-Luciferase-W vector. All of the PCR products were verified by sequencing (Genewiz). The lentiviral vectors pHAGE-EF1a-Cre-w^41^ and pHAGE-EF1a-mCherry-w were kindly gifted from Dr Darrell N. Kotton, Boston University. Lentiviral vectors were transfected into the packaging cell line HEK293 (ref. 41). After packaging the virus, supernatant was concentrated by ultracentrifugation to around 0.5–1 × 10^9^ PFU ml^−1^ titre. Lentiviral-mediated gene transduction was performed at the time of plating (Day −7 ALI) by infecting cells with lentivirus at ∼30 MOI in mTEC proliferation media supplemented with 5 μM of the Rho kinase inhibitor Y-27632 (Sigma)^41^,^42^, and washed with media a few days after virus transduction. Overall transduction efficiency was 30–60%, as judged by mCherry expression or indirect immunofluorescence using anti-HA-tag antibodies.

### Immunofluorescence and Immunohistochemistry

Lungs (embryonic, adult) were fixed in 4% paraformaldehyde (PFA) overnight at 4 °C; ALI cultures were fixed with 4% (PFA) for 10 min, room temperature or with 100% methanol for 20 min^41^,^42^. Human bronchial tissue sections were obtained from healthy non-identified donors. Immunostaining was performed on 5 μm paraffin lung sections (mouse, human) or on mouse ALI cultures, as described in refs 41, 42. Briefly, samples were incubated with primary antibodies for 2 h or overnight at 4 °C, washed in PBS and incubated with secondary antibody conjugated with Alexa488, 567 or 647 (Life Technologies, anti-Mouse: A21202, A10037, A31571, 1:300; anti-rabbit: A21206, A10042, A3157, 1:300) for 2 h. When necessary, antigen retrieval was performed using Unmasking Solution Tris-EDTA buffer (1 mM EDTA/Tris–HCL pH 8.3) for 15 min at 110 °C in a pressure cooker. The following antibodies were used: Anti-E2F4 (Millipore, LLF4.2, 1:100)^43^, anti-glutamylated tubulin (Adipogene, GT335, 1:250)^22^, anti-centrin (Millipore, 04-1624, 1:500)^22^, anti-centrin (Proteintech, 12794-AP-1, 1:500)^21^, anti-Pcm1 (Cell Signaling, #5213, 1:50; Santa Cruz, D-19, 1:50), anti-Scgb3a2 (ref. 33) (gift from Dr S. Kimura, NIH, 1:1000), anti-Foxj1 (ref. 45) (eBioscience, #2A, 1:100), anti-acetylated α–tubulin (Abcam, ab125356, 1:1000), anti-acetylated α–tubulin (Sigma T7451, 1:2000), anti-β-tubulin IV (Abcam, ab11315, 1:500), Anti-Cre (Millipore MAB3120, 1:100)^41^,^42^, and anti-Cre (Millipore, #69050, 1:200,; Cell signaling, #69050, 1:200)^41^,^42^, anti-HA (Cell signaling, #2367, #3724, 1:100)^41^, anti-C-Nap1 (Proteintech, 14498-1-AP, 1:100), anti-CEP63 (Proteintech, 16268-1-AP, 1:100)^21^. Anti-Deup1 (1:500), anti-CEP152 (1:500), anti-PLK4 (1:250) antibodies were produced in Dr Xueliang Zhu’s lab and previously published^21^. F-actin and nuclei were visualized by Alexa Fluor 647 phalloidin (Life tech. A22287, 1:100) and NucBlue Fixed Cell ReadyProbes Reagent (DAPI) (Life tech. R37606)^41^,^42^, respectively. To determine the specificity of signal from each antibody we used, as a negative control, normal rabbit, mouse or goat IgG (Santa Cruz) corresponding to the species of each primary antibody. The lack of epithelial signals in sections of *E2f4*^*+/f*^*; Shh*^*Cre/+*^ lungs served as an additional negative control for the specificity of the E2f4 antibody (LLF4.2) (Fig. 4b, Supplementary Figs 6a,b and 11). Further demonstration of specificity of the E2f4 antibody is provided in Supplementary Fig. 11. Images were acquired using a Nikon Labophot 2 microscope equipped with a Nikon Digital Sight DS-Ri1 charge-coupled device camera or on a Zeiss LSM 700 or LSM710 confocal laser-scanning microscope equipped with a Motorized Stage, an oil-immersion × 40 or × 63 objective lens and argon laser. For Z-stack analysis, scanning was performed at 0.25 μm per layer.

### 3D structured illumination (3D-SIM) microscopy

Structured illumination microscopy (SIM) was performed with a Nikon N-SIM based on an Eclipse Ti inverted microscope using an SR Apo-TIRF × 100/1.49 oil-immersion objective and an Andor iXon 3 EMCCD camera. Images were acquired in 3D-SIM mode using excitation at 488 nm and 561 nm and standard filter sets for green and red emission. Image z-stacks were collected with a z interval of 125 nm. SIM image reconstruction, channel alignment and 3D reconstruction were performed using NIS-Elements AR software.

### Morphometric analysis

For quantitative assessment of the changes in E2f4 subcellular localization we performed E2f4 immunostaining in airway epithelial cells isolated from WT adult tracheas cultured at ALI days 0, 2, 4 and 8 (as representative of stages 1, 2, 3 and 4, respectively). The subcellular localization of E2f4 was determined as signals present in the nucleus (overlapping with DAPI) or as cytoplasmic aggregates (>1 μm). The size of E2f4 granules (aggregates) was determined by measuring the longest diameter of the aggregate based on the signal intensity in each confocal image. Approximately 100 cells were counted per time point in five confocal Z-stack sections of three cultures and results were represented as percentage of E2f4-labelled cells with signals in nucleus or in cytoplasm (Supplementary Fig. 1). Moreover we analysed the temporal changes in cytoplasmic E2f4 in ALI cultures by counting the number of large cytoplasmic E2f4 granules (>1 μm) in 100 cells in 3 fields per time point at ALI days 0, 2, 4, 8. For the quantitative analysis of E2f4 association with early markers of centriole, we counted dot-like immunofluorescence signals of E2f4, Deup1, Plk4 or Cep152 in Z-stack images (0.25 μm per layer), typically 15–30 layers, in 3–5 non-overlapping random fields per experimental condition in confocal images captured at × 40 magnification (digital zoom of × 1.5– × 2.7). We analysed 10–50 cells for each marker based on nuclear staining marked by DAPI, using Zen software (Zeiss). To estimate how abundance of deuterosomes changes during multiciliogenesis we counted the total number of Deup1 dots per cell across confocal Z-stacks (0.25 μm per layer; 15–30 layers per cell) in ALI cultures from stages 2–4 (stage 1 has no deuterosomes) in 31 cells per group. Results were expressed mean (+s.e.) (Supplementary Fig. 3e). Changes in the proportion of deuterosomes present in Pcm1-enriched areas as a function of the number of deuterosomes per cell were determined by counting Deup1-Pcm1 double labelled dots (fully or partially overlapping signals were considered as positive) and the total number of Deup1 dots per cell across Z-plane stacks (15–30 layers per cell); the percentage of Deup1 double labelling (mean, +s.e.) was plotted against the number of deuterosomes per cell (in groups of 20) in 48 cells from 3 cultures. Similarly, we determined the number of Deup1 dots double labelled with centrin (partial and total overlap considered positive) in cells with different number of deuterosomes, and represented the results as described above (Supplementary Fig. 3f,g). For quantification of the diameter of Deup1-rings (cradles) we used Zeiss software (Zeiss, Blue edition) following reconstruction as 3D-SIM images^21^. Briefly, the Deup1 signal intensity peak was detected by profile analyses of each Deup1 rings (Zeiss software; see Supplementary Fig. 4b,c) and the diameters of Deup1-rings were calculated as follows: diameter= (*a*+*b*)/2; where *a*=longest diameter of a Deup1-ring and *b*=shortest diameter of Deup1-ring^21^ using 3D-reconstructed images. The presence of Deup1-rings either inside, at the periphery, or outside E2f4 granules (as represented in Fig. 2f, Supplementary Fig. 4c by *i,ii,iii* respectively, in the lateral panels) was determined at stage 3 (E2f4 granules >1 μm). We determined the relationship between size of deuterosome (diameter of Deup1 ring) and its position in the E2f4 granule (*i,* inside; *ii*, at the periphery, or *iii,* outside). If the highest signal intensity of the Deup1 ring found by profile analyses was detected inside or at the edge of the E2f4 granule, it was categorized as *(i) or (ii),* respectively, otherwise it was categorized as *(iii)*. Deup1 structures that still did not form a ring (typically those <200 nm) were ignored because small Deup1-dots were already identified as immature deuterosome components in a previous report^21^. We counted the number of cells in each category randomly in 10 cells at stage 3 and represented the results as the average size of deuterosome (mean ± s.e., nanometre diameter) in each group *(i, ii, iii).* Statistical analysis was performed (Student’s *t*-test) and differences were considered significant if *P<0.05.

### Quantitative real-time PCR

Total RNA from each sample was extracted using the RNeasy Mini Kit (Qiagen, #74104) and reverse-transcribed using Superscript III (Invitrogen, #18080-051)^41^,^42^. ABI 7000 (Applied Biosystems, Foster City, CA, USA) and Taqman probes for *E2f4*, *Plk4*, *Cep63*, *Deup1*, *Foxj1* and β*-actin* were used (Assays-on-Demand, Applied Biosystems, Austin, TX, USA). Reactions (25 μl) were performed using the TaqMan Gene Expression Assay (Applied Biosystems, TX, USA). The relative concentration of the RNA for each gene to beta-actin mRNA was determined using the equation 2–DCT, where D CT=(CT mRNA–CT β*−actin* R).

### Western blotting analysis

Cell extracts were prepared using a cell fractionation kit (Thermo Scientific, NE-PER Nuclear and Cytoplasmic Extraction Reagents) according to the manufacture’s protocol. For Western blotting^41^,^42^, samples were subjected to 4–12% gradient SDS-PAGE electrophoresis and transferred to PVDF membrane after quantification of the protein concentration via Bradford protein assay (Pierce Coomassie Plus (Bradford) Assay Kit from Thermo Scientific, Cat# 23236). Membranes were blocked with 8% (for E2f4 blot) or 5% (for all of other blots) Skim milk (2 h, R.T.) and probed with Anti-E2F4 (LLF4.2)(1:500)^43^, anti-Deup1 (1:3,000)^21^, Sas6 (91.390.21, Santa Cruz Biotechnology, 1:10). Anti-GAPDH (Cell signaling, #2118s, 1:5,000) and anti- LaminB1 (Abcam, ab65986, 1: 8,000) were used for the internal controls designating the cytoplasmic and nuclear fractions, respectively. Antigen–antibody complexes were identified wi\th HRP-conjugated secondary antibodies (Jackson lab). Enhanced chemiluminescence was detected using LAS 4000 (GE Health Care).

### Immunoprecipitation

293FT cells (Thermo Scientific, Cat# R70007) were transfected with with the following plasmids; pcDNA mouse E2f4, human SAS6 (Addgene #46382) or pcDNA-Flag-Deup1 using TransIT-LT1 reagent (Mirus). We also used mTECs at ALI day3 for endogenous protein interaction analyses. Cells were collected and lysed with NP40-based Lysis Buffer (NP-40 soluble fraction); 20 mM HEPES, 15% Glycerol (Thermo Scientific), 250 mM KCl, 1.2 mM EDTA (Thermo Scientific), 1% NP-40 (Thermo Scientific), Glutamax × 5 (Thermo Scientific), Proteinase & × 1 Phosphatase inhibitor (Thermo Scientific, Cat# 78443) and 0.1 mM Phenylmethanesulfonyl fluoride (PMSF) (Sigma Cat# P7626, add just before use from 200 mM stock) and 1 mM Sodium orthovanadate (200 mM stock) (Sigma Cat# S6508). The cell lysates were cleared by centrifugation at 3000G for 10 min at 4 °C. Immunoprecipitation for each samples (2 mg per sample) using 40 μl anti-E2F4 agarose beads (Santa Cruz, Cat# sc-866 AC) (prewashed with Lysis buffer to make 50% slurry) or anti-rabbit IgG agarose beads (Santa Cruz, Cat# sc-2345) as a control was carried out for 1–2 h on rocker on ice. After washing five times with cold 1 ml lysis buffer and centrifugation (168G, 1 min), 50 μl pH 2.5 Glycine buffer 2 × SDS loading buffer was added to elute protein complexes from each antibody. After gently tapping, these samples were centrifuged at 168G for 1 min at 4 °C. Each supernatant was transferred to another tube immediately to neutralize the pH to be 7.4 by adding 12ul pH8.5 Tris-HCL on ice. Then, approximately 12 μl × 6 SDS sample buffer (Reducing, Boston BioProducts, BP-111R) and DTT (final conc. 0.1 M) was added prior to boiling the samples for 10 min at 95 °C. Half of the samples (1 mg input) were loaded in a SDS-PAGE gel and westerns were performed as above. We used Mouse TrueBlot ULTRA: Anti-Mouse Ig HRP (Rockland, Cat# 18-8817-30, 1:1,000) to the E2F4 blots to decrease the signal of heavy chain. 80 μg per lane was loaded for the ALI culture input samples.

### Proximity ligation assay

PLA was performed according to the manufacturer’s protocol (Sigma, DUO92102)^46^. Briefly, ALI day 4 cultures *from E2f4*^*f/f*^*; R26*^*CreERT2/+*^ with or without Tamoxifen treatment were fixed with 4% PFA room temperature for 10 min. Samples were incubated with primary antibodies (anti E2f4, anti Deup1 or the relevant control IgGs, as indicated) using the immunofluorescence microscopy protocol described above, followed by incubation with the PLA probe set (anti-rabbit plus strand/anti-mouse minus strand) at 37 °C for 2 h. Ligation of probes in close proximity was carried out at 37 °C for 30 min, followed by a 100-min amplification step. Subsequently, we performed immunofluorescence staining for anti-Pcm1 antibody as described above. All processed slides were mounted using ProLong Gold antifade reagent (Life Technologies, catalogue no. P36930), and images were captured using a confocal microscope system (Carl Zeiss, LSM 710).

### Microarray analysis

Briefly, airway epithelial progenitors isolated from Tm or vehicle-treated cultures from adult *E2f4*^*f/f*^*; R26*^*CreERT2/+*^ tracheas were analysed at ALI days 0, 2 and 4 (representative of Stages 1, 2,3). Triplicate samples (each 2 wells per sample) of each group were incubated at 4 °C with RNA-later (Sigma) overnight at each time point (day 0, day 2, day 4). Total RNA was extracted from these samples (total 18) using a RNeasy Mini Kit (Qiagen, #74104). We used Mouse Gene 1.0 ST arrays; data were normalized using the Robust Multiarray Average (RMA) algorithm and a CDF (Chip Definition File) that maps the probes on the array to unique Entrez Gene identifiers. The technical quality of the arrays was assessed by two quality metrics: Relative Log Expression (RLE) and Normalized Unscaled Standard Error (NUSE). Pairwise Student *t* tests were performed between the control and mutant (KO) groups for comparisons within each time point. To identify centriole biogenesis genes differentially altered in *E2f4* mutant (KO) cultures and control, we first examined the expression of 724 genes associated with centriole biogenesis reported in Ma *et al*. (Table 1 of reference^24^). This revealed expression of 266 of these genes significantly altered at D0 in *E2f4* KO cultures. Clustering analysis showed that 10 genes are clustered together with *Deup1/Ccdc67* (downregulated in KO samples at ALI day0) and 16 genes are clustered together with *Plk4* (downregulated in KO samples at day2). This contrasted with the relatively unchanged levels of Cep63 (consistent with results from Ma *et al*.^24^. Changes in gene expression were confirmed by qRT-PCR.

### Data availability

All relevant data are included in this article and Supplementary Information files or available from the authors upon request. Microarray datasets have been deposited in the NCBI GEO database (GEO Submission No. GSE73331).

## Additional information

**How to cite this article:** Mori, M. *et al*. Cytoplasmic E2f4 forms organizing centres for initiation of centriole amplification during multiciliogenesis. *Nat. Commun.* **8,** 15857 doi: 10.1038/ncomms15857 (2017).

**Publisher’s note:** Springer Nature remains neutral with regard to jurisdictional claims in published maps and institutional affiliations.

## Supplementary Material

Supplementary Information

Supplementary Movie 1

Supplementary Movie 2

Supplementary Movie 3

Supplementary Movie 4

Supplementary Movie 5
