# Author Correction: Cytoplasmic E2f4 forms organizing centres for initiation of centriole amplification during multiciliogenesis

**DOI:** 10.1038/ncomms16157

**Published:** 2018-11-19

**Authors:** Munemasa Mori, Renin Hazan, Paul S. Danielian, John E. Mahoney, Huijun Li, Jining Lu, Emily S. Miller, Xueliang Zhu, Jacqueline A. Lees, Wellington V. Cardoso

Nature Communications 8: Article number: 15857; DOI: 10.1038/ncomms15857 (2017); Published 07 04 2017; Updated 11 19 2018.

This Article contains an error in reference 1. The correct reference is as follows:

Sánchez, I. & Dynlacht, B. D. Cilium assembly and disassembly. *Nat. Cell Biol.* **18**, 711–717 (2016).

