# Supplementary material for: Cytoplasmic E2f4 forms organizing centres for initiation of centriole amplification during multiciliogenesis
Source: Nat Commun. 2017 Jul 4;8:15857. doi: 10.1038/ncomms15857 (PMC5500891; doi:10.1038/ncomms15857)
Supplement: Supplementary Information [file ncomms15857-s1.pdf]

SI GUIDE

Type of file: pdf
Size of file: 0 KB
Title of file for HTML: Supplementary Information
Description:

Type of file: MP4
Size of file: 0 KB
Title of file for HTML: Supplementary Movie 1
Description: E2f4 is localized at the core of Pcm1-containing granules: Computergenerated reconstruction of images obtained from super resolution 3D-structured illumination microscopy showing top and side views of E2f4-Pcm1 granules in ALI day 2 cultures of airway epithelial progenitors. Pcm1 (in red) is consistently present at the surface of these granules while E2f4 (in green) is at its core.

Type of file: MP4
Size of file: 0 KB
Title of file for HTML: Supplementary Movie 2
Description: Deuterosome assembly in E2f4-enriched regions at stage2: 3D-SIM imaging: Deup1 (red), E2f4 (green). Larger Deup1-rings are found outside or adjacent to E2f4-granules.

Type of file: AVI
Size of file: 0 KB
Title of file for HTML: Supplementary Movie 3
Description: Deuterosome assembly in Pcm1-enriched regions at stage2: Confocal microscopy, maximum projection 3D-reconstructed view: immature deuterosomes (Deup1, red small dots) shown inside Pcm1 regions while mature decorated with multiple centrin dots (green) are seen outside Pcm1 regions (blue). Nucleus: DAPI (grey).

Type of file: AVI
Size of file: 0 KB
Title of file for HTML: Supplementary Movie 4
Description: Deuterosome maturation at stage2~3: Confocal microscopy, maximum projection 3D-reconstructed view: abundant mature deuterosomes (Deup1, red) decollated multiple centrin dots (green) are seen outside Pcm1 (blue). Nucleus: DAPI (grey).

Type of file: MP4
Size of file: 0 KB
Title of file for HTML: Supplementary Movie 5
Description: Centriole amplification at stage3~4: confocal microscopy, maximum projection 3D-reconstructed view: Late stage cells with abundant centrin labeling (green; prospective basal bodies) and multiple mature deuterosomes (Deup1, red) outside the Pcm1 (blue). Nucleus: DAPI (grey).

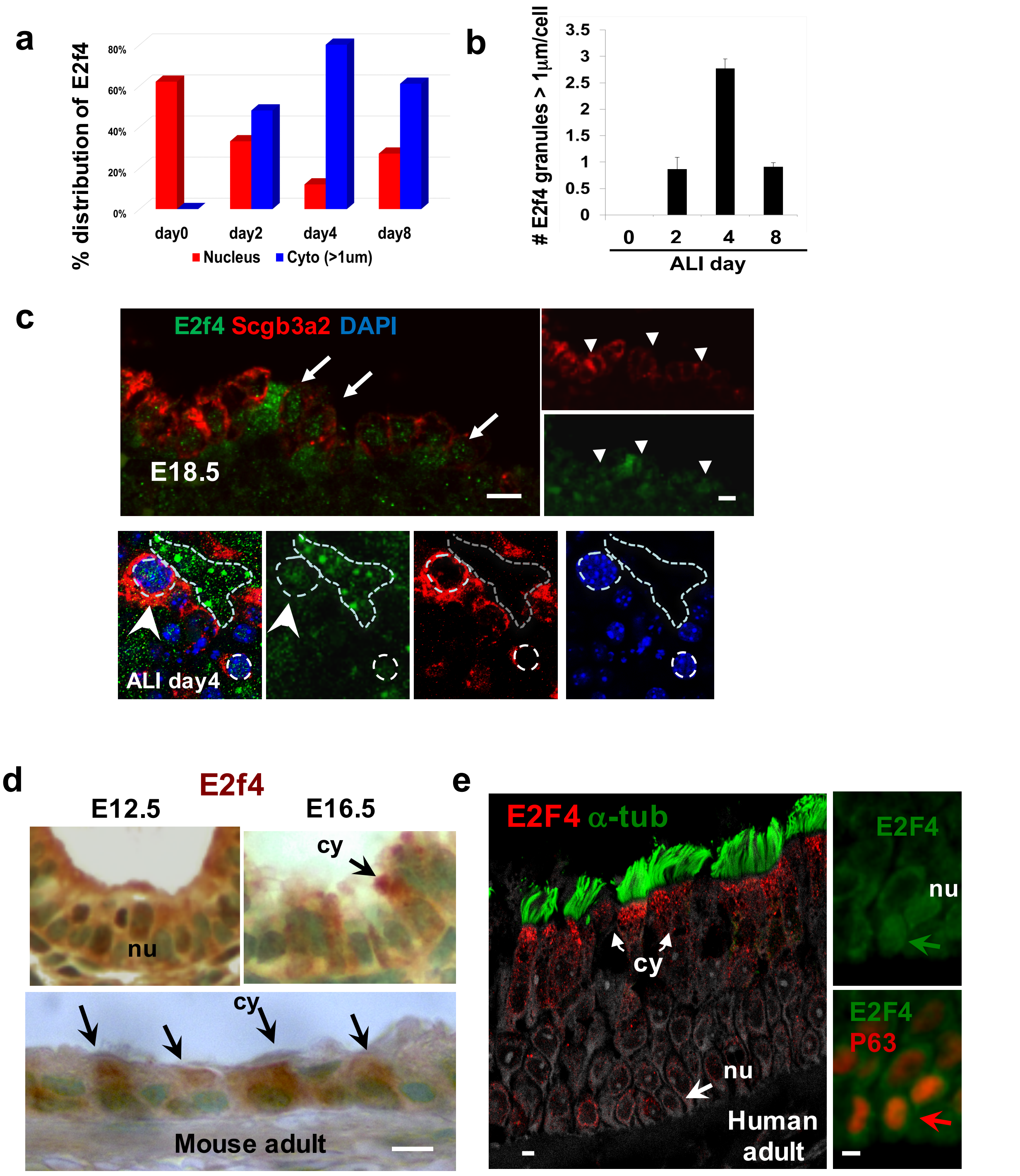

Supplementary Fig. 1. Distribution of nuclear and cytoplasmic E2f4 in developing and adult airway epithelium. (a) Dynamic changes in E2f4 subcellular localization: quantitative analysis, immunofluorescence (IF) of ALI days 0,2,4,8 cultures as representative of stages 1, 2, 3 and 4,

respectively. Bars: percentage E2f4-labeled cells with signals only in nucleus (red) or as cytoplasmic aggregates (>1μm, blue) in five confocal Z-stacks (n = 100 cells per time point). (**b**) The number of large cytoplasmic E2f4 granules (>1μm) per cell increases from ALI day 0-4 (bars: mean ±se of 100 cells in 3 fields per time point). **(c)** E2f4 IF-confocal imaging: nuclear signals in differentiating secretory cells (Scgb3a2-labeled); panels: *top*: E18.5 lung (arrows: nuclear signals); *bottom* ALI day4: E2f4-DAPI in nuclei of secretory cells (dotted areas; compare with cytoplasmic E2f4 granules in neighbor cell) likely contributing to the population of cells expressing nuclear E2f4 at ALI day 8 (depicted by red bar in **a**); (**d**) E2f4 immunoperoxidase (DAB) staining in developing and adult murine airways: cytoplasmic (cy) and nuclear (nu) signals; nuclei counterstained with methyl green. **(f)** E2F4 IF in adult human airway: strong cytoplasmic signals in multiciliated cells (labeled by acetylated α-tub); nuclear E2F4 in basal cells (nu, arrows in left and right panels identified by E2F4-P63 double-labeling). Images in all panels representative of n> 3 samples per group. All bars= 10μm.

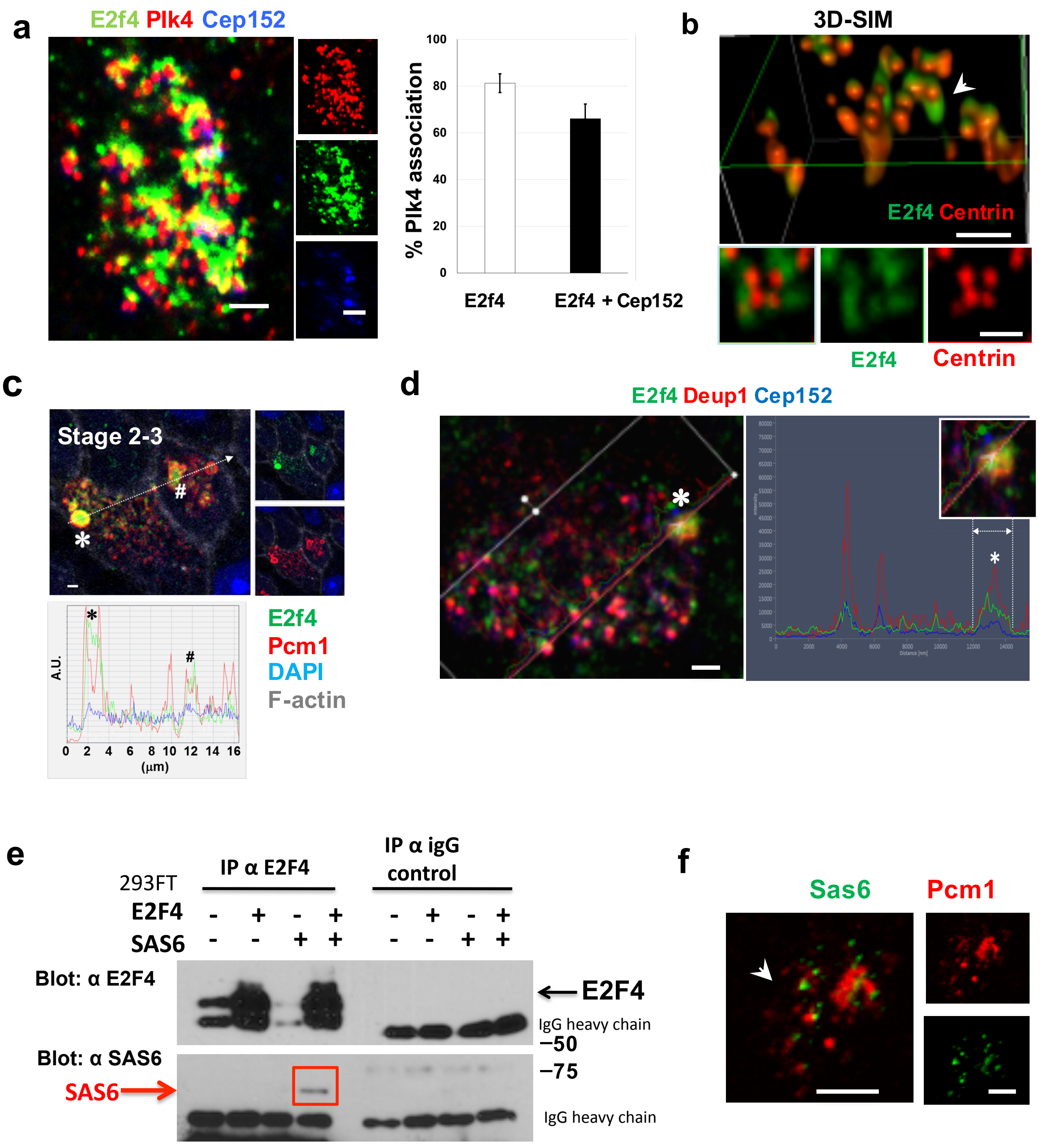

Supplementary Fig. 2. Cytoplasmic E2f4 associates with Pcm1 and early centriole biogenesis markers during initiation of centriole amplification (Stage 2-3 cells). (a) Confocal image of apical aggregates triple-labeled with E2f4, Cep152 and Plk4 at stage 2; graph depicts percentage of Plk4-labeled dots overlapping with E2f4 or E2f4-Cep152 double-labeled dots in Z-stack images (bars are mean ±se of measurements in 3-5 non-overlapping random fields per condition, n= 3

cultures, 0.25μm/layer). **(b)** 3D-SIM: E2f4 association with centrin (arrowhead and cradle-like structure in lower panels). **(c)** E2f4 at the core or Pcm1 granules in stage 2-3 cells: E2f4-Pcm1 double IF, colocalization shown by assessment of signal intensities across a line in representative confocal section (Graph: A.U., arbitrary unities; distance in micrometers**;** peaks depicted as * and # in two cells outlined by F-actin and stained with DAPI). (**d)** E2f4, Pcm1, Deup1 overlap confirmed by profile analysis of signal intensity across a representative confocal section in stage 2-3 cells. Graph: arbitrary units (AU) (large granule highlighted by * also shown in inset, right panel areas) **(e)** Immunoprecipitation, Western blot analysis: binding of E2f4 to Sas6 in 293FT cells transfected with mouse E2f4 and human SAS6. **(f)** Sas6-accumulation in Pcm1-enriched areas of stage 2 cells. Bars: a, b, c, d, f=2, 0.5, 1, 1, 2.5 μm, respectively.

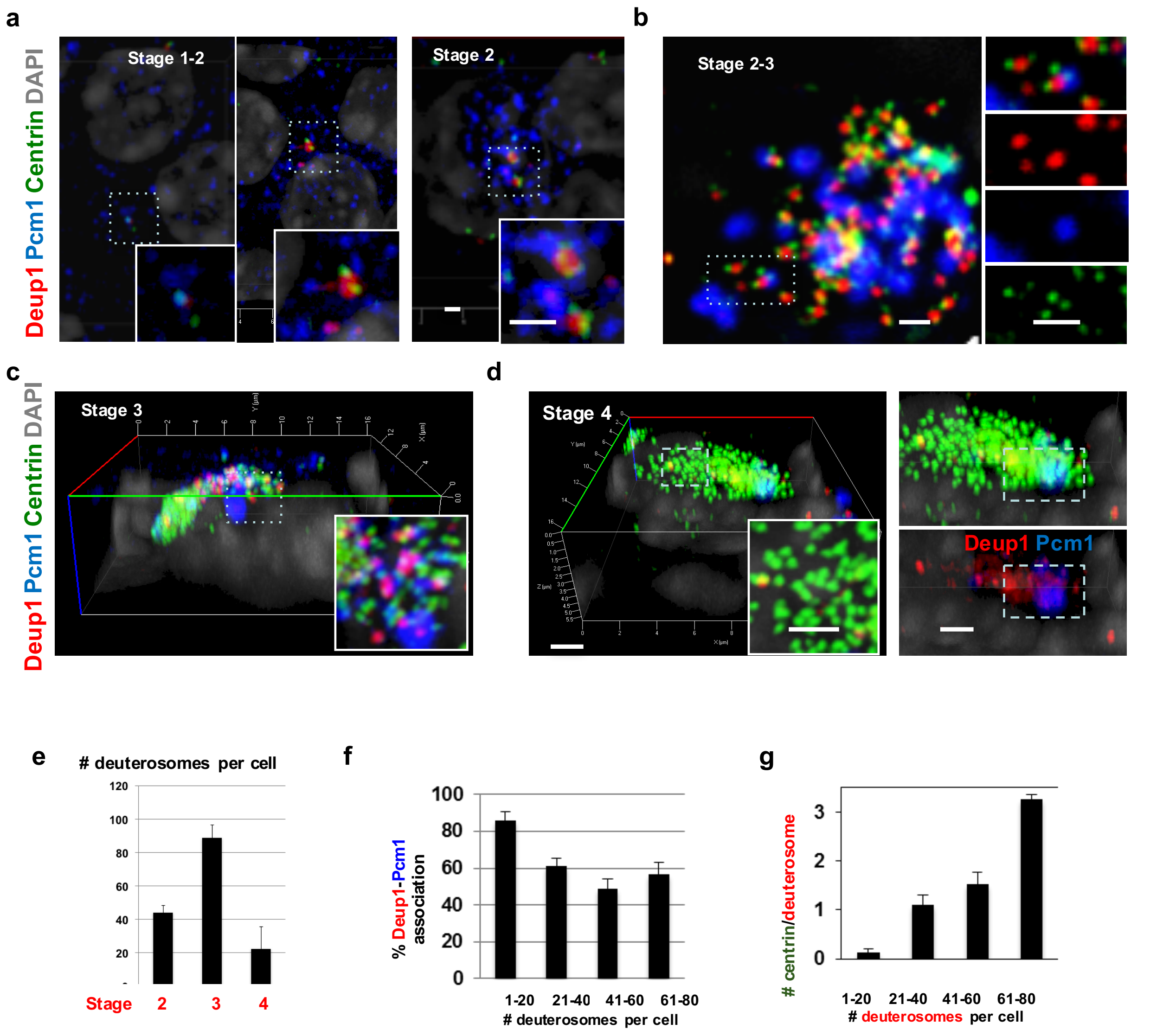

Supplementary Fig. 3. Spatial-temporal relationship between deuterosomes assembly and Pcm1-labeled granules (FG) during centriole amplification. Confocal images and 3D reconstruction depicting Deup1, Pcm1, Centrin during deuterosome assembly and centriole amplification in airway progenitors in Stages 1-4 cells (representative images of 32 cells from three replicate cultures per stage). **(a)** Scattered Deup1-labeled dots in cells transitioning from stage 1-2 become increasingly associated with Pcm1-enriched areas (also enriched in E2f4, see Fig. 2a, c) and initiate assembly and nucleation of deuterosomes (insets depict centrin-decorated Deup1 dots). **(b)** Stage 2 to 3 transition: as the deuterosomes' number, size and association with

centrins increase they become more frequently found at the periphery or outside Pcm1 areas (mature deuterosomes, see also Fig. 2f and Suppl. Fig. 4b, c) **(c, d)** At late stage 3 and 4 cells show abundant centrin labeling largely outside the Pcm1 regions and dissociated from deuterosomes, (inset: boxed areas). (**e)** Dynamic changes in number of deuterosome per cell at Stages 2,3,4 cultures: bars: mean +se of 31 cells per group (n =3 replicates) counted in confocal Z-stacks (15-30 layers per cell). (**f)** Changes in the proportion of Deup1 dots double-labeled with Pcm1 (deuterosomes in Pcm1-enriched areas) as the number of deuterosomes per cell increases (number of Deup1 dots /cell from 1 to 80 grouped as depicted): bar: mean (+se) of 3 replicates. **(g)** Increasing number of deuterosomes decorated with centrins as the number of deuterosomes per cell increases. Graph: bars (mean+se) represent number of Deup1 dots double-labeled with centrin in cells with 1-80 deuterosomes. Bars: a, b, d = 0.5, 2, 2μm respectively.

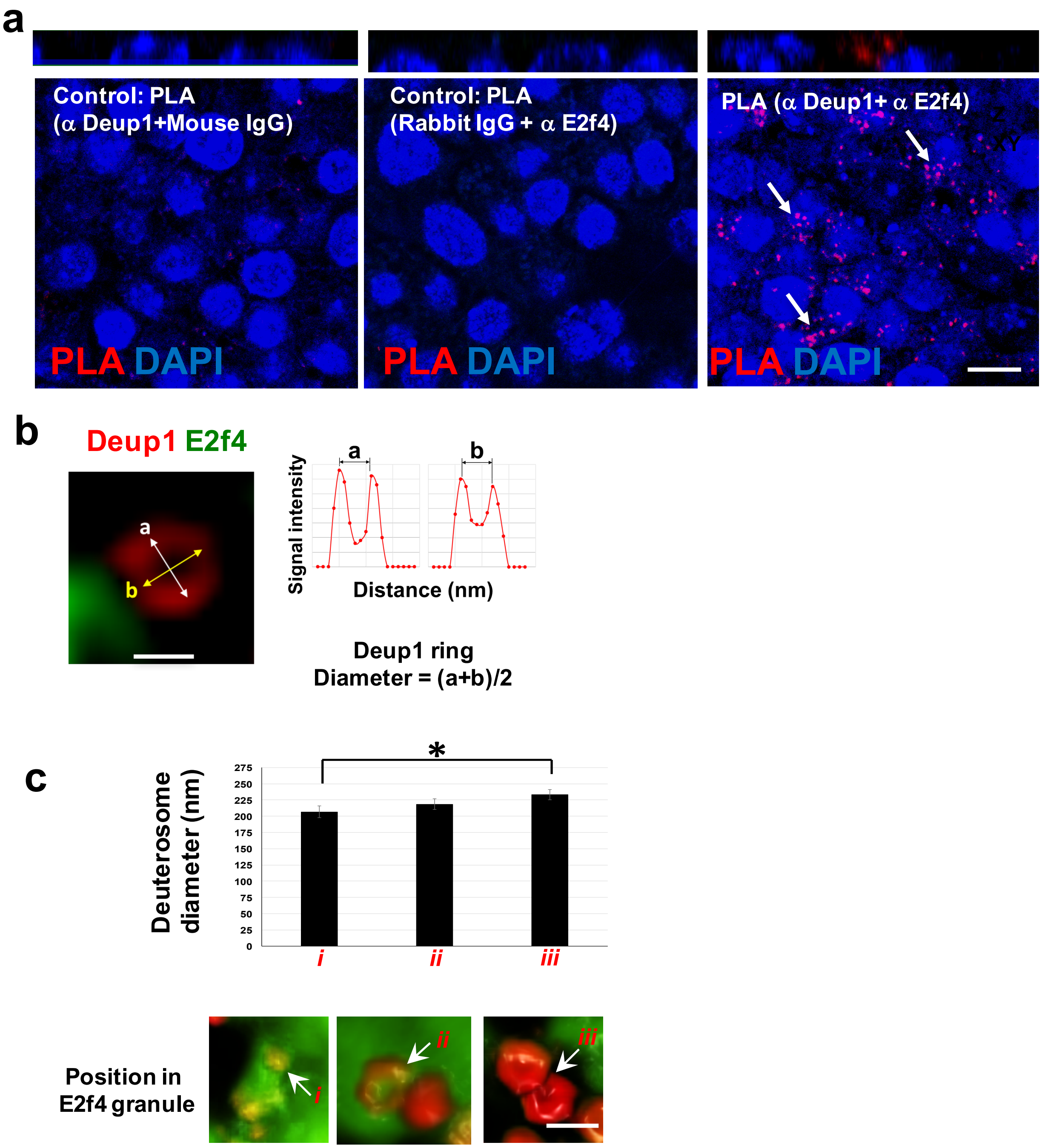

Supplementary Fig. 4. Deup1-E2f4 association during deuterosome assembly and maturation. (**a**) Proximity ligation assay (PLA) to detect proximity of Deup1 and E2f4 proteins (within 40nm of each other) in stage 2-3 cells (ALI day 2). Representative panels of quality control experiments to support results in Fig. 2d (from n=3 per experimental condition). PLA signals

detected in multiple cells using combined specific antibodies against Deup1 and E2f4. Specificity confirmed by absence of PLA signals in the presence of each antibody plus IgG control antibodies (mouse or rabbit). **(b, c**) Quantitative assessment of deuterosome diameters and relationship between deuterosome size and position in the E2f4 granules. Deup1-E2f4 immunofluorescence and 3D-SIM reconstruction in Stage 2 and early stage 3 cells. **(b)** Deuterosome size as determined by profile analyses of Deup1 signal intensity (distance between peaks in nanometers) across the highest and smaller diameters (depicted as *a* and *b*) and estimated as *a*+*b*/2 (see methods). **(c)** Deuterosome size and relative position in the E2f4 granules: graph depicts mean +se of deuterosome diameters (nanometers) in regions of total *(i*), partial *(ii)* or no *(iii)* Deup1-E2f4 overlap counted in 57 deuterosomes (total) from samples represented in Fig. 2f. Individual panels (below) correspond to each of the bars in the graph. Statistical analysis was performed (Student's t-test) and differences were considered significant if $p < 0.05$* Bars: a, b, c = 5, 0.5, 0.5, respectively.

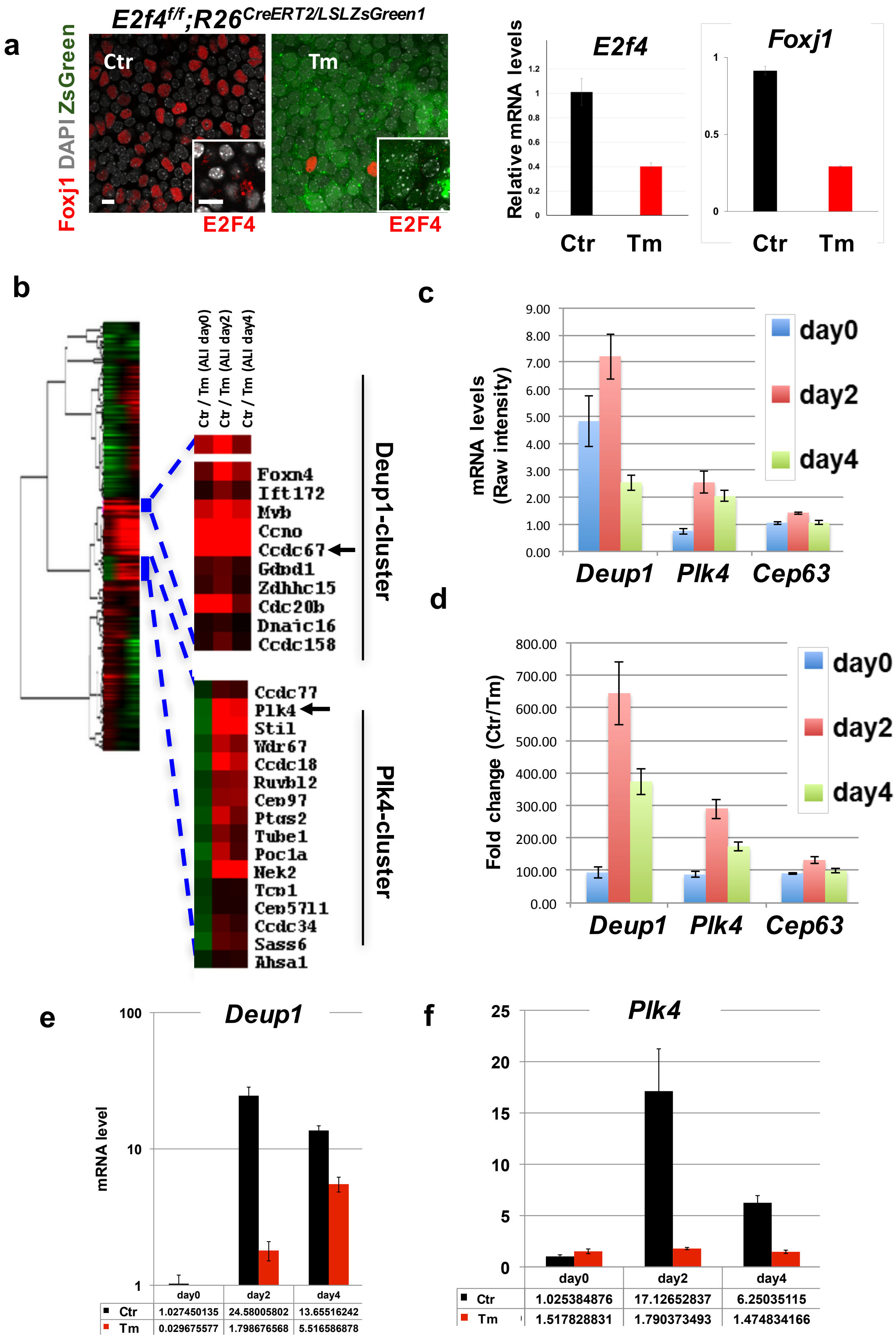
a
E2f4f/f;R26CreERT2/LSLZsGreen1
Foxj1 DAPI ZsGreen
Ctr
Tm
E2F4
E2F4
Relative mRNA levels
E2f4
Foxj1
Ctr Tm
Ctr Tm
b
Ctr / Tm (ALI day0)
Ctr / Tm (ALI day2)
Ctr / Tm (ALI day4)
Foxn4
Ift172
Mvb
Ccno
Ccdc67
Gdnd1
Zdhhc15
Cdc20b
Dnaic16
Ccdc158
Deup1-cluster
Ccdc77
Plk4
Stil
Wdr67
Ccdc18
Ruvbl2
Cep97
Ptgs2
Tube1
Poc1a
Nek2
Tcp1
Cep57l1
Ccdc34
Sass6
Ahsa1
Plk4-cluster
c
mRNA levels (Raw intensity)
day0
day2
day4
Deup1 Plk4 Cep63
d
Fold change (Ctr/Tm)
day0
day2
day4
Deup1 Plk4 Cep63
e
Deup1
mRNA level
day0 day2 day4
Ctr 1.027450135 24.58005802 13.65516242
Tm 0.029675577 1.798676568 5.516586878
f
Plk4
day0 day2 day4
Ctr 1.025384876 17.12652837 6.25035115
Tm 1.517828831 1.790373493 1.474834166

Supplementary Fig. 5. Nuclear function of E2f4 in regulating transcription of centriole biogenesis genes in murine adult airway progenitors. (**a**) Confocal imaging immunofluorescence for Foxj1 and E2f4 (inset) in ALI day 4 control vehicle (Ctr) and Tm-treated adult $E2f4^{f/f}$; $R26^{CreERT2/LSLZGreen1}$ cells. Disruption of multiciliogenesis in Tm-treated progenitors with significant reduction in E2f4 and Foxj1 expression, confirmed by qPCR (Bars, mean ±se; n=3 cultures per group; efficient Cre-mediated recombination as shown by *LSLZsGreen1)*. (**b**) Heatmap, microarray analysis of $E2f4^{f/f}$;$R26^{CreERT2/LSLZGreen1}$ of ALI days 0, 2, 4 cultures (Ctr and Tm, n=3 per group). *Deup1 (Ccdc67*) and *Plk4* clusters identified by gene enrichment analysis and comparison with E2f4-Multicilin targets reported in *Xenopus* (see methods) [24] (**c, d**) Distinct temporal regulation of E2f4 target genes: *Deup1* cluster downregulated at ALI day0, *Plk4* cluster downregulated at ALI day2. *Cep63* was unchanged. **(c)** Bars represent mean (±se) mRNA levels of *Deup1*, *Plk4* and *Cep63* in each group based on raw intensity of hybridization in microarrays; (**d**) Bars represent mean (±se) fold-change values (Ctr/Tm) for each gene and time point. **(e, f)** qPCR of *Deup1* and *Plk4* at ALI days 0, 2 and 4 (samples used for microarray) confirming the temporal expression seen in microarray analysis, bars represent mean (±se) mRNA levels . Bar: 10μm.

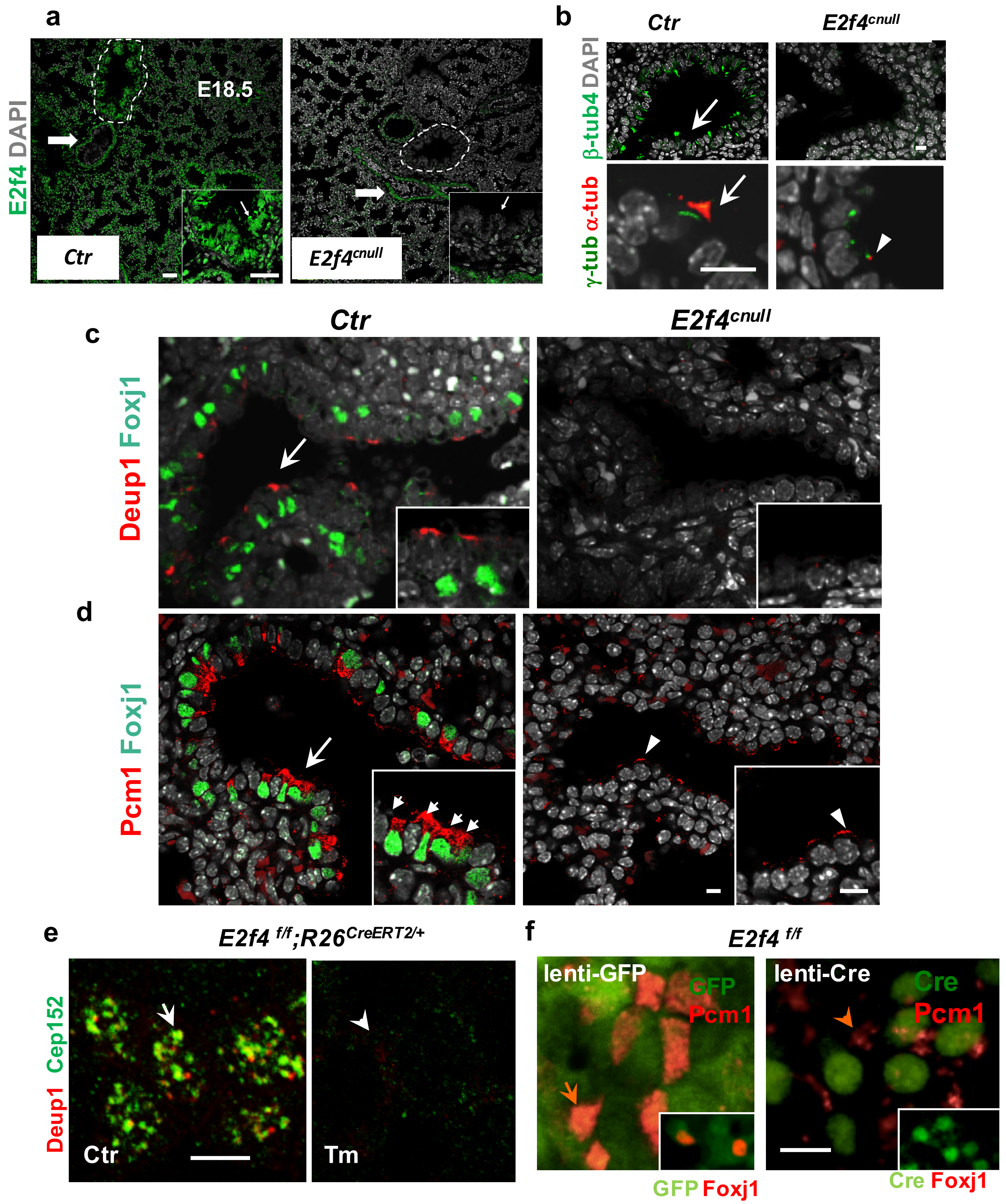

Supplementary Fig. 6. Inability to form deuterosomes and Pcm1-containing aggregates in E2f4-deficient epithelium in vivo and in vitro. (a-d) Immunofluorescence analysis of E18.5 *E2f4*^{cnull} and control (Ctr, *E2f4* $^{f/f}$) lungs. **(a)** Selective disruption of E2f4 expression in the

developing lung epithelium of *E2f4*^{cnull} (*Shh* ^{Cre}*; E2f*$^{f/f}$) mice. Note preserved E2f4 expression in the vascular compartment not targeted by *Shh* ^{Cre} driver (large arrow) in contrast to the absence of signals in epithelium of airways (dotted area; small arrow in insets) and distal lung**. (b-d)** Inability of *E2f4*^{cnull} to form multiciliated cells (marked by β-tubulinIV, γ-tubulin, acetylated α-tubulin, Foxj1 in controls) but preserved primary cilia (**b**, arrowhead: α-tub and γ-tub). **(c)** Deup1 expression in multiciliated cells of E18.5 control lungs (arrow in Foxj1 positive cells, enlarged in inset) is lost in *E2f4*^{cnull} (right panel: no Deup1 or Foxj1), consistent with the inability to form deuterosomes and initiate multiciliogenesis. **(d)** The strong Pcm1 staining in Foxj1-expressing cells of controls (arrow) is markedly decreased in *E2f4*^{cnull} lungs (arrowheads, insets; residual Pcm1 expression likely due to E2f4-independent regulation). **(e, f)** E2f4 deletion in ALI cultures of Tm-treated *E2f4* $^{f/f}$; *R26* $^{CreERT2/+}$ or lentiviral *Cre*- transduced *E2f4*$^{f/f}$ cells confirms the major decline in Deup1, Pcm1 and Cep152 signals (arrows and arrowheads depict signals in control and mutants, respectively; insets in g illustrate inability of Cre-labeled cells to induce Foxj1 expression). Bar: a, b-f, 50, 10μm, respectively.

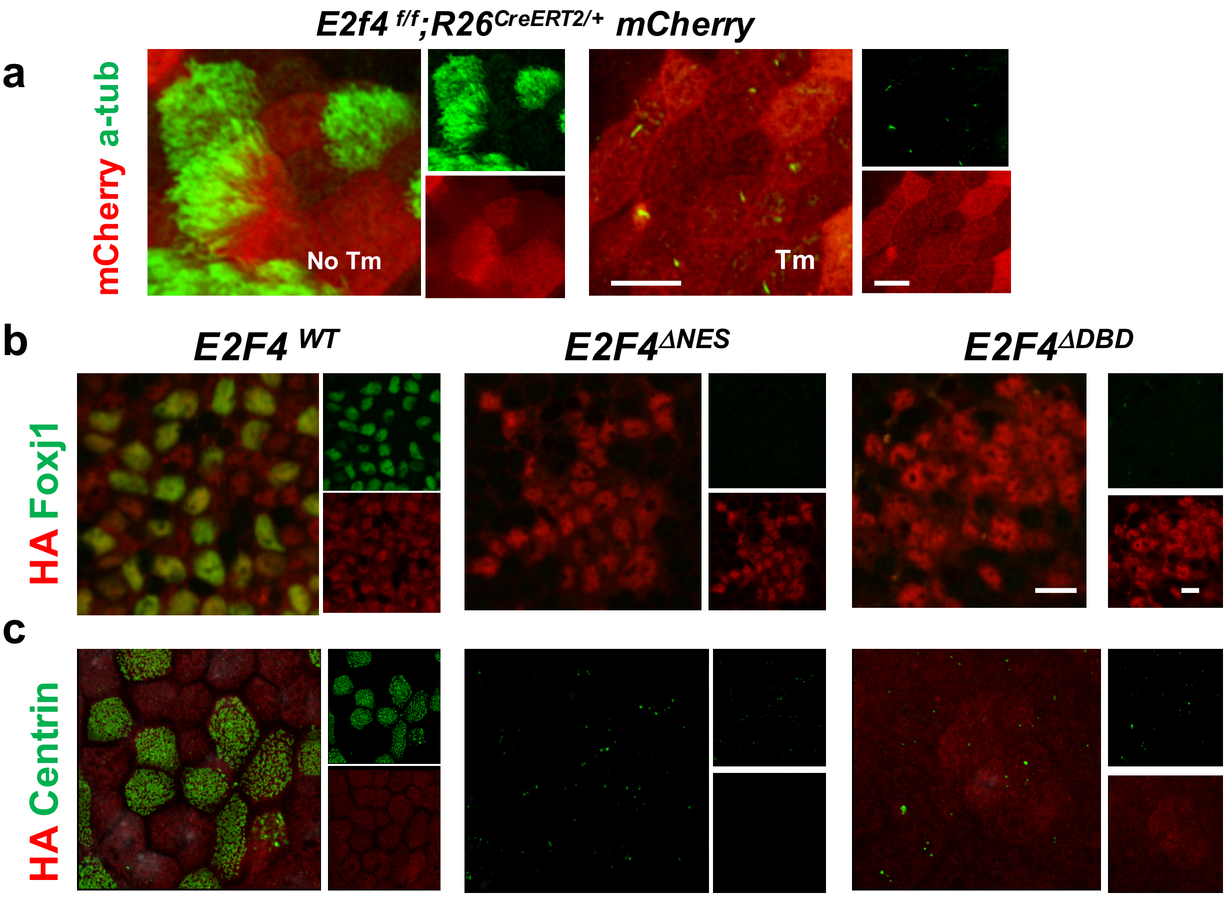

Supplementary Fig. 7. Impact of E2f4 subcellular localization in centriole amplification and commitment to multiciliogenesis in epithelial progenitors. IF/confocal imaging. **(a)** Lentiviral-mediated induction of mCherry negative control or E2F4-HA constructs in $E2f4^{f/f}$; $R26^{CreERT2/+}$ airway progenitors. Preserved multiciliogenesis (α-tub in multicilia) in the absence of Tm (Tamoxifen), while only primary cilia were present in Tm-treated cultures. (**b, c**) rescue of Foxj1 and centrin expression in Tm-treated $E2f4^{f/f}$;$R26^{CreERT2/+}$ cells transduced with $E2F4^{WT}$ but not with $E2F4^{\Delta NES}$ or $E2F4^{\Delta DBD}$. Bars: 5µm

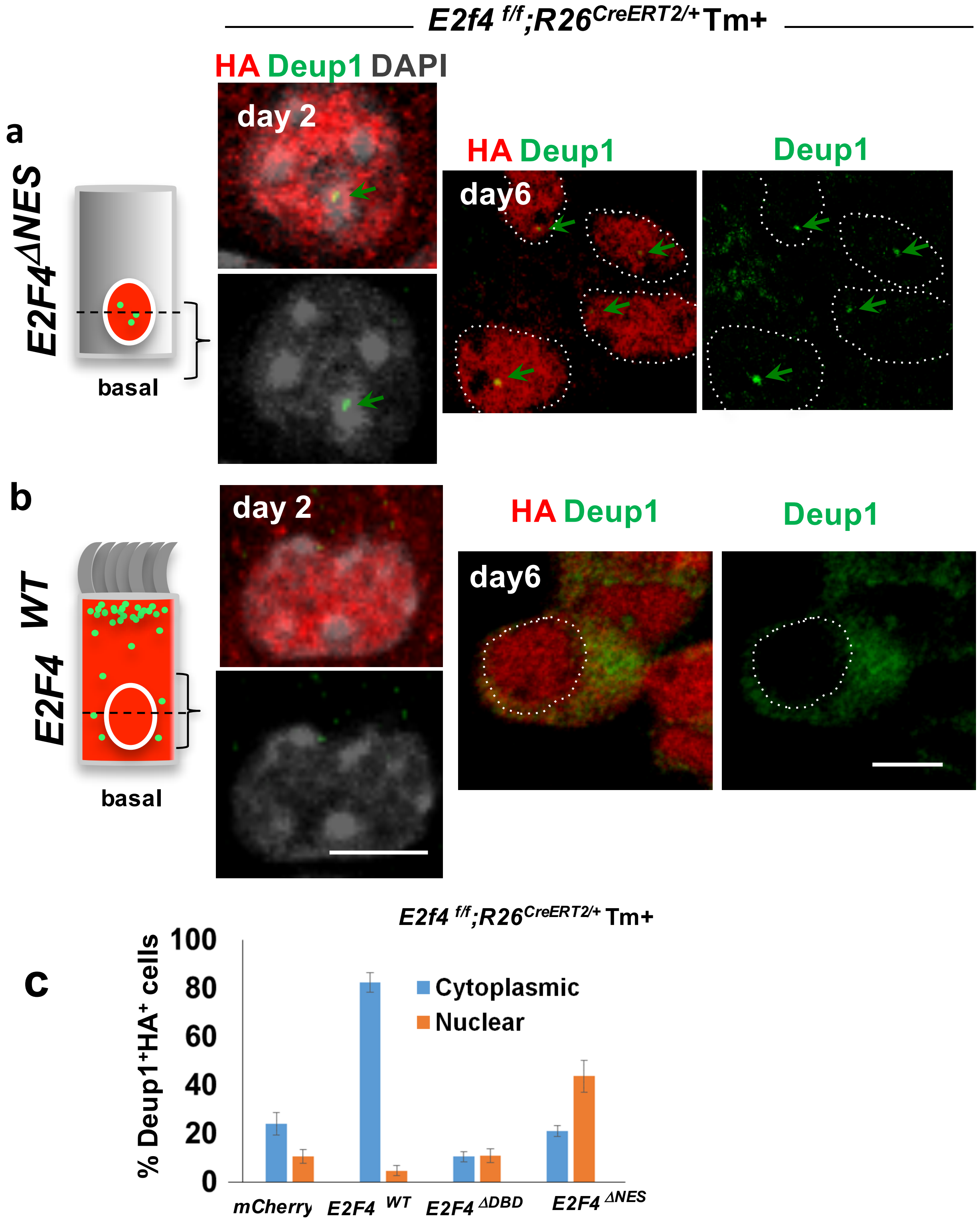

Supplementary. Fig. 8. Cytoplasmic E2f4 is required for proper subcellular localization of Deup1. (a-c) Confocal imaging: HA-Deup1 immunofluorescence for HA, Deup1, DAPI depicting

solely the basal-lateral aspect of Tm-treated *E2f4* $^{f/f}$;*R26* $^{CreERT2/+}$ cultures at day 2 and day 6 transduced with *E2F4*$^{\Delta NES}$ **(a)** or *E2F4*^{WT} (**b**) constructs (for apical expression please see Fig 3-4)**.** *E2F4*$^{\Delta NES}$ rescues Deup1 but protein is mislocalized in nuclei (arrows; nuclei marked by DAPI in left panels and represented in dotted areas in right panels); **(c)** *E2F4*^{WT} rescues Deup1 expression (apical Deup1 cytoplasmic aggregates depicted in Fig.3) and prevents the nuclear mislocalization seen in *E2F4*$^{\Delta NES}$ **(b).** Quantitative analysis of the subcellular distribution of Deup1 in cells transduced with each E2F construct in Tm-treated *E2f4*$^{f/f}$; *R26* $^{CreERT2/+}$cells. Note major difference between *E2F4*$^{\Delta NES}$ and in *E2F4*^{WT} cells. Bars are mean $\pm$se of the percentage of HA +cells with Deup1 labeling in cytoplasm or nucleus counted in 5 different fields in three independent experiments. All bars: 5μm

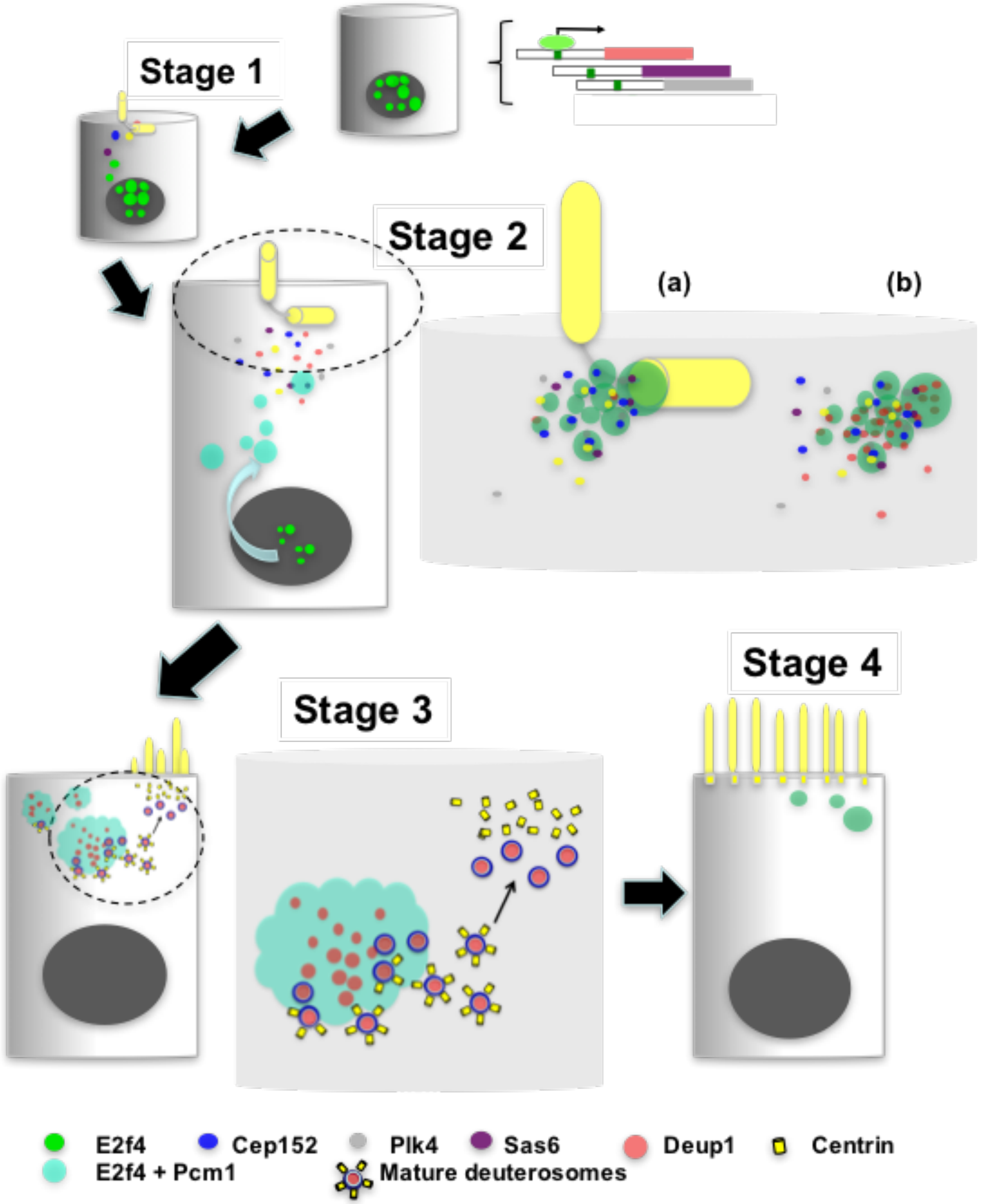

Supplementary Fig. 9. Schematic illustrating the E2f4-mediated events in multiciliogenesis.

(Stage 1) Nuclear E2f4 in multiciliated cell precursors initiates a transcriptional program of

centriole biogenesis genes. **(Stage 2)** E2f4 shuttles to the cytoplasm where it associates with Pcm1 to become a core component of apical granules (fibrous granules) at the centrosomal (*a*) and nearby regions (*b*). E2f4-Pcm1 granules act as organizing centers for recruitment of centriole biogenesis proteins (Deup1, Cep152, Plk4, Sas6, centrin) and initiate assembly of deuterosomes. **(Stage 3)** Deuterosomes become abundant and increasingly decorated with centrins (mature deuterosomes) and become progressively less associated with the Pcm1-E2f4 granules. **(Stage 4)** Large-scale centriole production is achieved and is followed by apical centriole docking (basal body) and multicilia formation.

Fig. 1b

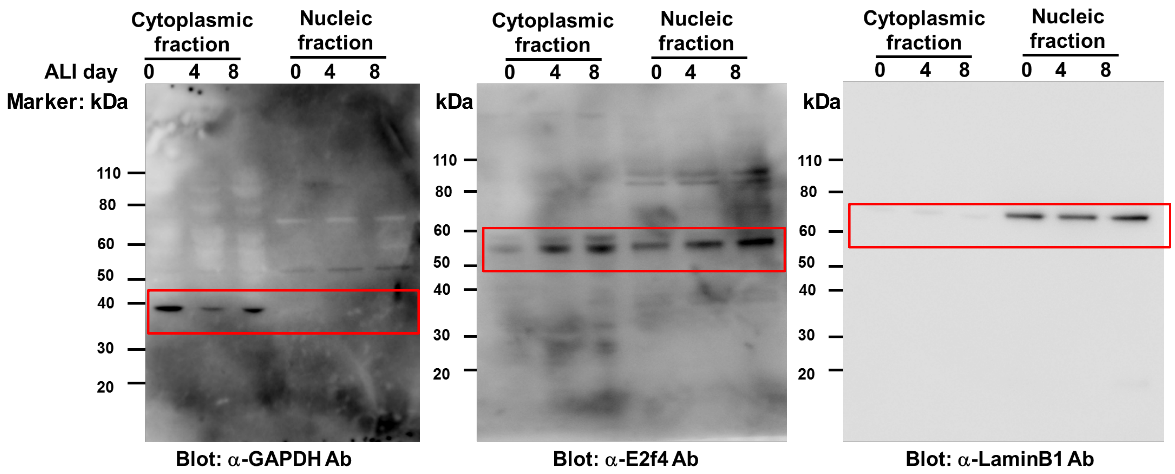

Fig. 2e

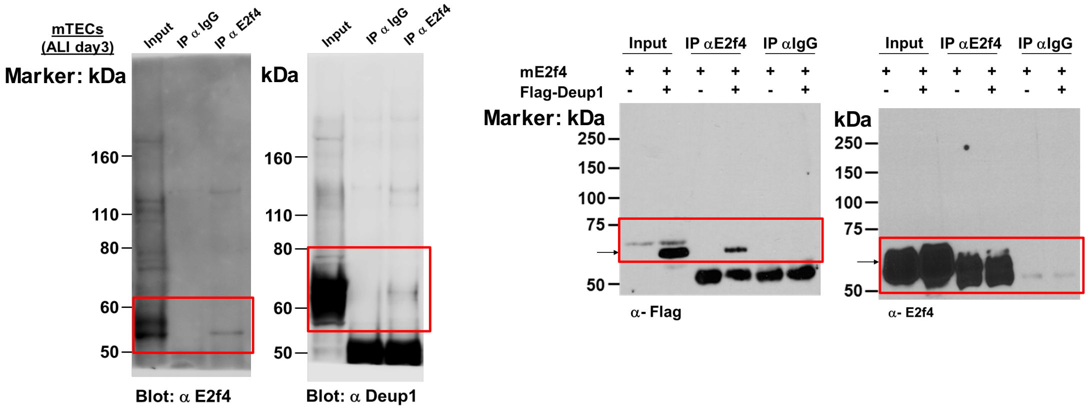

Suppl. Fig. 2d

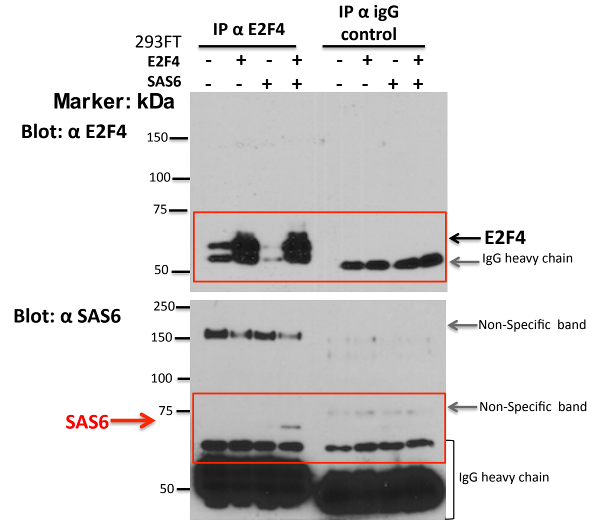

Supplementary Fig. 10. Raw data files of Western blot analyses: Full scans of original blots.

The boxed regions indicate the areas shown in the figures.

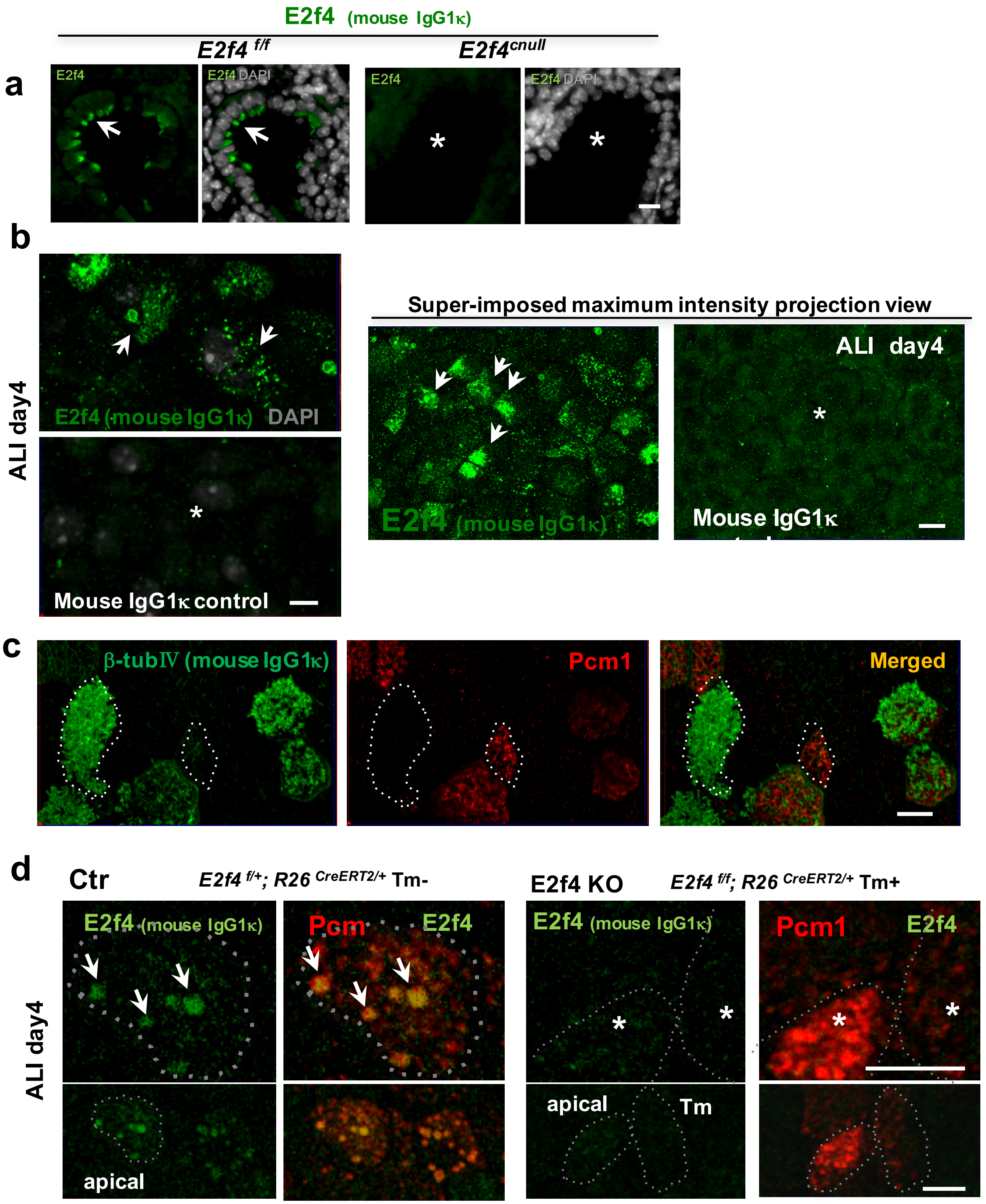

Supplementary Fig. 11. Demonstration of specificity of the E2f4 antibody used ins all IF assays (LLF4.2 mouse IgG1κ; please see also methods). **(a)** Immunostaining with anti-E2f4

antibody is completely abolished in E2f4 null cells of E18.5 *E2f4*^{cnul} lungs. Strong E2f4 signals in the epithelium of E2f4 sufficient (left panels: arrow in E*2f4* $^{f/f}$, DAPI) but not in *E2f4*^{cnull} lungs (right panels; also in Suppl. Fig. 6). **(b)** No signals are detected by immunostaining of WT cells with the isotype control IgG1κ antibody. Immunofluorescence analysis of ALI day4 cultures (WT) using the anti-E2f4 antibody (LLF4.2 mouse IgG1κ) shows the characteristic cytoplasmic E2F4 granules (arrows) but no signals in cultures stained with the mouse IgG1κ isotype control antibody (asterisk). Specificity further confirmed by confocal analysis showing no signals in IgG1κ control even in a super-imposed maximum intensity projection XY view of Z-stacked images (right panels). **(c)** Additional proof of specificity is provided indirectly by demonstrating that other mouse IgG1κ antibodies, such as that against β-tubulinIV shows a pattern of expression very distinct from that of E2f4 (LLF4.2) in WT ALI day 4 cultures, including minimal to no overlap with Pcm1 (dotted areas). **(d)** The E2f4 antibody is not binding non-specifically to fibrous granules as detected by Pcm1. Left panels: immunofluorescence staining of control (*E2f4* $^{f/+}$*; R26* $^{CreERT2/+}$*)* ALI day 4 cultures with antibodies against E2f4 (LLF4.2) and Pcm1 showing the expected overlapping pattern in large cytoplasmic granules (arrows). Right panels: The E2f4 pattern does not result from LLF4.2 reacting non-specifically with other proteins present in Pcm1-labeled granules. *E2f4* $^{f/f}$*; R26*$^{CreERT2/+}$ *c*ells were plated and initially expanded under control conditions; cultures were then subjected to a late exposure to Tm (from 2 days prior to induction of ALI to ALI day2) and analyzed at ALI day4. The initial expression of E2f4 allowed Pcm1 accumulation, which remained present at various levels in these cells once E2f4 was permanently deleted. Double-IF E2f4 (LLF4.2)-Pcm1at ALI day revealed no E2f4 signal in E2f4 null cells regardless the abundance of Pcm1 in these cells (asterisks show cells expressing high and low levels of Pcm1 side-by-side). This further confirmed the specificity of the E2f4 patterns identified by the LLF4.2

antibody. For additional demonstration please see Fig. 4b showing overlap between HA and E2f4 (LLF4.2) IF signals in Tm-treated *E2f4* $^{f/f}$; *R26* $^{CreERT2/+}$ cultures transduced with *E2F4*^{WT}. Bars: a, b-d =10, 5μm, respectively.
